# Structure–Property Relationships in Symmetrical Bolaamphiphilic Dehydrodipeptides: Self-Assembled Injectable Hydrogels for Anticancer Drug Delivery

**DOI:** 10.3390/gels12040306

**Published:** 2026-04-03

**Authors:** Carolina Amorim, André Carvalho, Pedro R. Figueiredo, Alexandra T. P. Carvalho, Loic Hilliou, David M. Pereira, Helena S. Azevedo, José A. Martins, Paula M. T. Ferreira

**Affiliations:** 1 Chemistry Centre, University of Minho (CQ-UM), 4710-057 Braga, Portugal; carolinaamorim753@gmail.com (C.A.); id9569@alunos.uminho.pt (A.C.); 2 CNC—Center for Neuroscience and Cell Biology, Institute for Interdisciplinary Research (IIIUC), University of Coimbra, 3004-504 Coimbra, Portugal; pmrfigueiredo@cnc.uc.pt (P.R.F.); apirescarvalho@gmail.com (A.T.P.C.); 3 Almac Sciences, Department of Biocatalysis and Isotope Chemistry, Almac House, 20 Seagoe Industrial Estate, Craigavon BT63 5QD, UK; 4 Institute for Polymers and Composites, University of Minho, 4800-058 Guimarães, Portugal; loic@dep.uminho.pt; 5 REQUIMTE/LAQV, Laboratório de Farmacognosia, Departamento de Química, Faculdade de Farmácia, Universidade do Porto, R. Jorge Viterbo Ferreira, n 228, 4050-313 Porto, Portugal; dpereira@ff.up.pt; 6 i3S—Instituto de Investigação e Inovação em Saúde, Universidade do Porto, Rua Alfredo Allen, 208, 4200-180 Porto, Portugal; hazevedo@i3s.up.pt; 7 INEB—Instituto de Engenharia Biomédica, Universidade do Porto, Rua Alfredo Allen, 208, 4200-180 Porto, Portugal

**Keywords:** bolaamphiphilic dehydropeptides, self-assembly, supramolecular hydrogels, self-healing hydrogels, drug delivery

## Abstract

Peptide-based supramolecular hydrogels have emerged as promising biomaterials due to inherent biocompatibility, tunable self-assembly, and structural similarity to the extracellular matrix. This work describes the design, synthesis and characterization of a library of symmetrical bolaamphiphiles based on dehydropeptides, systematically varying both the dehydroamino acid residue and the linker. Aromatic and aliphatic dicarboxylic acids with distinct rigidities were employed to elucidate their influence on molecular self-assembly, hydrogelation, and functional performance. Hydrogel formation was triggered using a pH-responsive approach, and critical aggregation and gelation concentrations were determined. Morphological analysis by transmission electron microscopy revealed dense fibrillar networks with nanometer-scale fiber diameters, while rheological studies demonstrated viscoelastic behavior, tunable mechanical strength, and, in selected systems, efficient self-healing properties. The incorporation of phenylalanyldehydrophenylalanine significantly enhanced hydrogel formation, highlighting the importance of π–π interactions and hydrophobicity. Biological evaluation using HaCaT keratinocytes confirmed low cytotoxicity across the series. A representative injectable hydrogel exhibited sustained release of the anticancer drug methotrexate, governed predominantly by Fickian diffusion. These results establish clear structure–property–function relationships and demonstrate the potential of symmetrical bolaamphiphilic dehydropeptides as versatile platforms for controlled drug delivery.

## 1. Introduction

Self-assembly is a fundamental process underlying the formation of hierarchical structures in living systems and has inspired the development of supramolecular materials for biomedical applications [1,2,3,4,5,6,7]. In this context, peptide-based hydrogels have attracted significant interest due to their intrinsic biocompatibility, biodegradability, and ability to mimic the extracellular matrix at both the structural and functional levels [8,9,10].

Short peptides are capable of self-assembling into a wide range of nanostructures, including fibrils, tapes, nanotubes, and vesicles, driven by non-covalent interactions such as hydrogen bonding, hydrophobic interactions, and π–π stacking [5,11,12,13,14,15,16,17]. Among these materials, low-molecular-weight peptide hydrogelators are particularly attractive because they combine molecular simplicity with structural tunability and responsiveness to external stimuli such as pH, temperature, and ionic strength [18,19,20,21,22].

Bolaamphiphiles are amphiphilic molecules that feature two hydrophilic head groups, one at each end, linked by a hydrophobic spacer. [13,23,24,25]. Peptide-based bolaamphiphiles offer distinct structural features compared to *N*-capped peptides, particularly their symmetric architecture, which can promote well-defined and controllable self-assembly behavior [26]. Symmetrical bolaamphiphiles incorporating dehydropeptides have recently emerged as efficient supramolecular hydrogelators, combining enhanced proteolytic stability with strong self-assembly propensity [27,28,29].

Previous studies from our group demonstrated that symmetrical bolaamphiphilic *bis*-dehydropeptides form stable hydrogels with promising mechanical properties and selective drug release behavior [29,30]. However, the influence of linker length and rigidity and dehydroamino acid composition on hydrogel formation and function has not been systematically explored. Addressing this gap is essential for the rational design of bolaamphiphilic peptide-based hydrogels with tailored mechanical and biological performance [31]. In this work we will explore the hydrogelation of a series of bolaamphiphiles based on dehydropeptides with several aromatic and alkyl linkers.

## 2. Results and Discussion

### 2.1. Molecular Design and Synthesis

**Figure 1:**
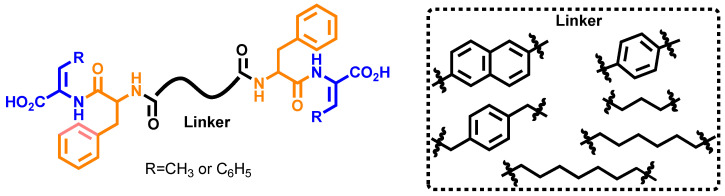
General structure of dehydropeptide-based symmetrical bolaamphiphiles explored in this work.

Symmetrical bolaamphiphiles were synthesized using solution-phase peptide chemistry, following protocols established by the research group for the synthesis of dehydropeptides [29,30,32] (Supporting Information, Schemes S1 and S2) (Figure 1).

**Figure 2:**
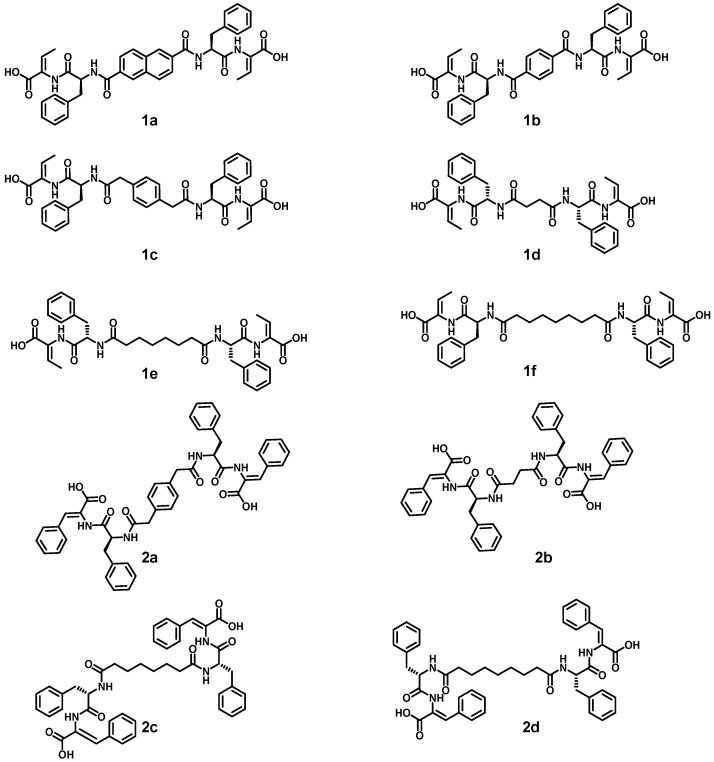
Structure of dehydropeptide-based bolaamphiphiles explored in this work.

Dehydropeptide blocks, phenylalanyldehydrophenylalanine (H-*L*-Phe-Z-∆-Phe-OH) and phenylalanyldehydroaminobutyric acid (H-*L*-Phe-Z-∆Abu-OH), were deployed to get insight into the interplay between aromaticity and hydrophobicity of the dehydropeptide block on the self-assembly behavior of the bolaamphiphiles. A series of aromatic and aliphatic dicarboxylic acid linkers, including naphthalene-2,6-dicarboxylic acid, terephthalic acid, 2,2′-(1,4-phenylene)diacetic acid, succinic acid, suberic acid, and azelaic acid, were employed to systematically modulate molecular flexibility (Figure 2). This design strategy enabled the evaluation of π–π interactions *versus* van der Waals and hydrophobic interactions in driving supramolecular assembly.

### 2.2. Self-Assembly and Hydrogelation

#### 2.2.1. Critical Aggregation Concentration

**Figure 3:**
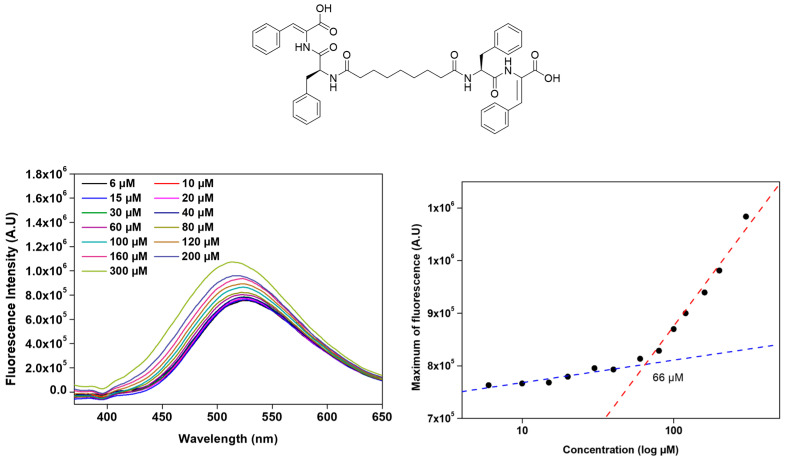
Illustrative example, with peptide **2d** of the ANS methodology used in this work to determine the critical aggregation concentration of dehydropeptide-based bolaamphiphiles.

The critical aggregation concentration (*CAC*) of the bolaamphiphilic dehydrodipeptides was calculated using 8-anilinonaphthalene-1-sulfonate ammonium salt (ANS) as an extrinsic fluorescence probe or the intrinsic fluorescence of the bolaamphiphile [33,34,35]. ANS is weakly fluorescent in polar aqueous environments but undergoes a pronounced enhancement in fluorescence intensity, accompanied by a blue shift in the emission maximum, upon association with hydrophobic domains on molecular aggregates. Fluorescence emission spectra of ANS (*λ*_exc_ = 370 nm) were acquired as a function of increasing peptide concentration. The *CAC* was determined from a plot of ANS maximum fluorescence intensity *versus* peptide concentration. The *CAC* parameter was taken as the intersection of the linear trend observed for the low peptide concentration range, corresponding to the dispersed state, and the high-concentration regime, associated with aggregate formation (Figure 3, Table 1) (Supporting Information, Figure S1).

The *CAC* values calculated for the bolaamphiphilic dehydrodipeptides are within the concentration range 39–153 µM, revealing a rich relationship between the bolaamphiphiles’ molecular structure and their self-assembly propensity (Table 1). Bolaamphiphiles incorporating the L-Phe-Z-∆Phe-OH motif (**2a**–**d**) consistently exhibited greater hydrophobicity than the corresponding L-Phe-Z-∆Abu-OH derivatives (**1a**–**f**), resulting in aggregation at lower concentrations and highlighting the dominant role of hydrophobic interactions in driving self-assembly in aqueous media (Table 1). In contrast, bolaamphiphiles incorporating the L-Phe-Z-∆Phe-OH dehydropeptide motif (**2a**–**d**) exhibited only a weak correlation between aggregation concentration and hydrophobicity. Moreover, for the dehydropeptides (**1a**–**f**) containing the block *L*-Phe-Z-∆-Abu-OH, the aggregation concentration increases moderately with cLogP. Interestingly, compounds **1a** and **1c**, featuring aromatic linkers, show aggregation concentrations similar to those seen for compounds **2a**–**d** despite significantly lower hydrophobicity. Strikingly, compounds **1c** and **2a**, sharing the same aromatic linker, display similar aggregation propensity despite significantly different hydrophobicity. The aromatic linker on compounds **1c** and **2a** seems to promote efficient molecular packing, overriding the effect of hydrophobicity on self-assembly. Long, flexible alkyl linkers likely introduce entropic penalties on molecular aggregation and suboptimal aromatic alignment, resulting in higher *CAC* values. Overall, the aggregation propensity of the dehydropeptide-based bolaamphiphiles is not determined by hydrophobicity alone, but there is a complex interplay between the molecular geometry structure of the dehydropeptide block and linker aromaticity, rigidity and flexibility, and molecular hydrophobicity [9].

#### 2.2.2. Molecular Dynamic Simulations

**Figure 4:**
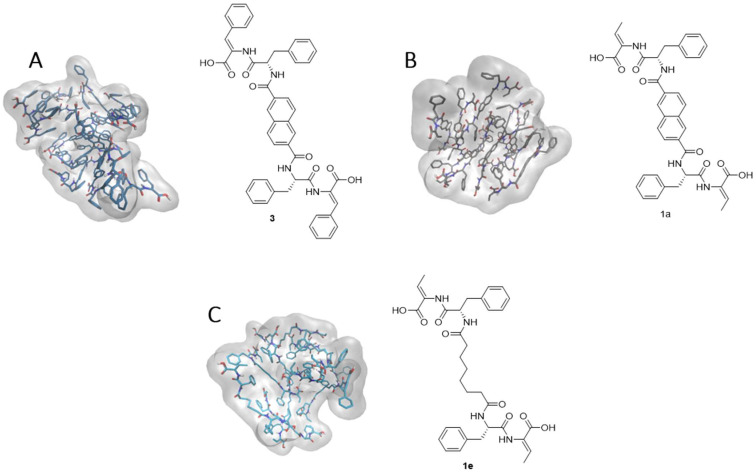
Snapshots from the most frequent MD simulation structure, displaying the self-assembled architecture of compounds **3** [29] (**A**), **1a** (**B**) and **1e** (**C**). For simplicity, water molecules and non-polar hydrogens were omitted. Representative molecular interactions observed for these compounds are supplied in the Supporting Information.

Molecular dynamics (MD) simulations were performed to investigate the influence of the dehydroamino acid residue and linker on self-assembly behavior. Atomistic MD simulations were conducted for the previously reported compound **3** [29], as well as compounds **1a** and **1e**. Atomistic MD simulations were carried out to understand the interactions that led to the architecture of hydrogels of the previously described compound **3** [29] (Figure 4) and of compounds **1a** and **1e** in water. For each system, 10 randomly dispersed units were solvated with an orthorhombic box of water molecules. Following energy minimization, the systems were heated to 310 K, equilibrated, and then simulated for 300 ns in an isothermal–isobaric ensemble (Figure 4). Representative intra- and intermolecular interactions are supplied in the Supporting Information (Figures S2–S4).

In compound **3**, extensive intermolecular π–π interactions were observed between the naphthalene linkers, as well as between the naphthalene moieties and the ΔPhe aromatic ring, predominantly through sandwich and parallel-displaced stacking arrangements. These interactions resulted in a highly interconnected supramolecular network. In contrast, the aromatic rings from Phe exhibited only weak intra- and intermolecular interactions, although occasional contacts with the naphthalene linker were observed. Substitution of the dehydrophenylalanine residue in compound **3** with dehydroaminobutyric acid (compound **1a**) significantly reduced intermolecular linker–linker interactions, leading to the formation of more compact, spherical aggregates. This system displayed fewer intermolecular π–π interactions, which occurred mainly between the Phe aromatic ring and the naphthalene moiety through T-shaped and, less frequently, parallel-displaced arrangements. Similar to compound **3**, the Phe side chain in compound **1a** contributed weakly to the overall interaction network. Replacement of the aromatic linker with an aliphatic chain (compound **1e**) resulted in a substantial decrease in intermolecular interactions. In this case, π–π stacking was limited to occasional contacts between Phe aromatic rings, and only a small number of hydrogen bonds were observed throughout the simulation. Consequently, compound **1e** formed loosely packed, spherical aggregates, reflecting the reduced extent of stabilizing intermolecular interactions (Figure 4). These findings are consistent with the experimentally determined critical aggregation concentrations (*CAC*s), with compound **1e** exhibiting a significantly higher *CAC* than compound **1a** (153 µM vs. 51 µM, respectively; Table 1), indicative of weaker self-assembly propensity.

#### 2.2.3. Hydrogelation

**Figure 5:**
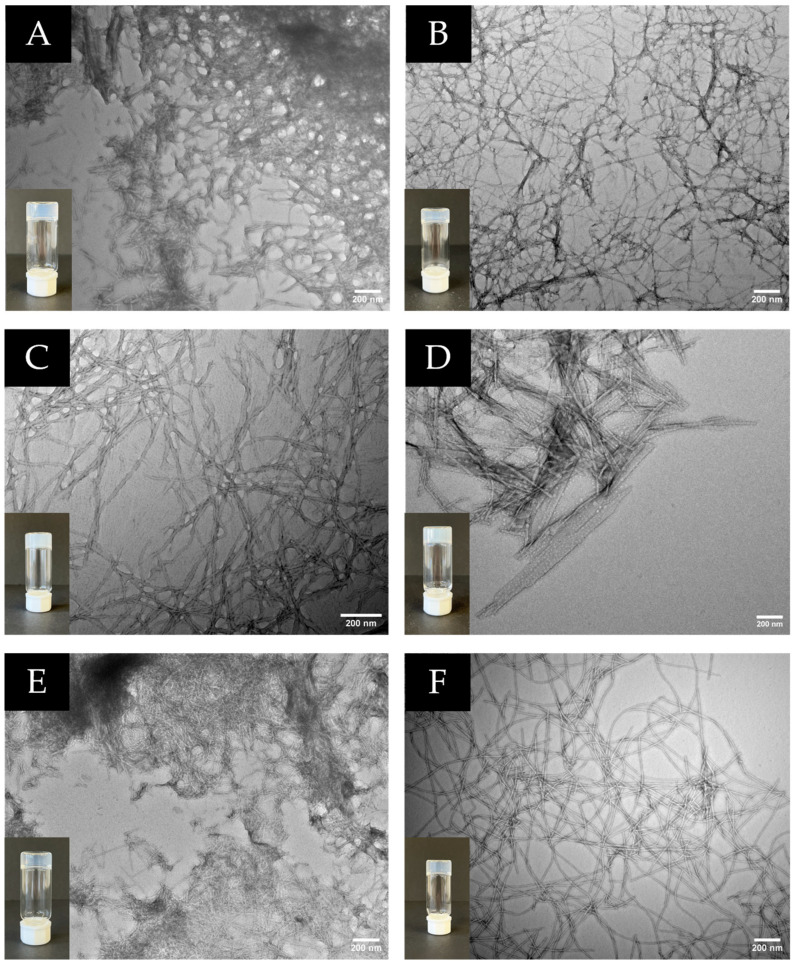
Hydrogels and transmission electron microscopy of hydrogels from compounds **1c** (**A**), **1e** (**B**), **2a** (**C**), **2b** (**D**), **2c** (**E**), **2d** (**F**).

Hydrogelation of the dehydropeptide-based bolaamphiphiles was accomplished using the pH-triggered approach, by addition of glucono-δ-lactone (GDL), which affords homogeneous supramolecular hydrogels (Table 2, Figure 5) [32,36]. The pH change was obtained by the aqueous hydrolysis of GdL into D-gluconic acid. All bolaamphiphiles featuring the dehydropeptide *motif L*-Phe-Z-∆Phe-OH (**2a**–**d**) afford hydrogels, but only compounds **1c** and **1e** in the dehydropeptides’ series *L*-Phe-Z-∆-Abu-OH originate hydrogels. With the exception of compound **1e** (*CAC* = 153 µM), the gel forming bolaamphiphiles (**1c**, **2a**–**d**) display CAC values bellow 86 µM, affording gels at 0.4 wt% concentration. Bolaamphiphile **1e** gels are afforded at 0.5 wt% concentration. Higher propensity for supramolecular aggregation (lower *CAC* values) seems to translate into effective formation of fibrillar hydrogel networks. Interestingly, compound **1a**, with a *CAC* value similar to **1c**, failed to originate hydrogels, whilst peptide **1e**, despite displaying much higher CAC, afforded hydrogel. Compound **1a** features a rigid aromatic linker, while compounds **1c** and **1d** bear aromatic and alkyl linkers, with higher flexibility. This result highlights the importance of linker flexibility for molecular aggregation, supramolecular packing and network buildup.

Transmission electron microscopy revealed that all hydrogels consist of dense, interconnected fibrillar networks. Fiber diameters ranged from approximately 8 to 20 nm for most systems, while selected compositions displayed mixed fibrillar and lamellar morphologies. These nanoscale architectures are consistent with the formation of physically cross-linked three-dimensional networks (Figure 5).

Optical microscopy images were obtained to further investigate the self-assembly behavior of the compounds that do not form hydrogels (Figure S5, Supporting Information). Compound **1a** showed amorphous aggregates while **1b** formed well-defined needle-like crystalline structures indicating that crystallization prevails over gelation. Bolaamphiphile **1d** displayed long fibrillar structures, although forming a weak network insufficient for hydrogel stabilization. In contrast, **1f** presented spherical aggregates, suggesting the formation of micelle- or vesicle-like structures rather than a fibrillar network.

#### 2.2.4. Rheological Properties of the Hydrogels

The rheological behavior of the hydrogels was evaluated at their critical gelation concentrations (CGC) to elucidate gelation kinetics, mechanical strength, yielding behavior, and recovery. Hydrogels from boalamphiphiles **1e** and **2b** exhibit rapid gelation, with the storage modulus (G′) exceeding the loss modulus (G″) almost immediately after sample loading, indicating fast network formation. In contrast, hydrogels **1c** and **2a** displayed pronounced lag times (≈15,000–25,000 s) before sol-to-gel transition, indicating slower aggregation and network build-up. Hydrogelators **2c** and **2d** also gelled more slowly, with elastic behavior appearing only after around 15,000 and 10,000 s, respectively, reflecting weaker or more gradual assembly pathways (Supporting Information Figure S6).

**Figure 6:**
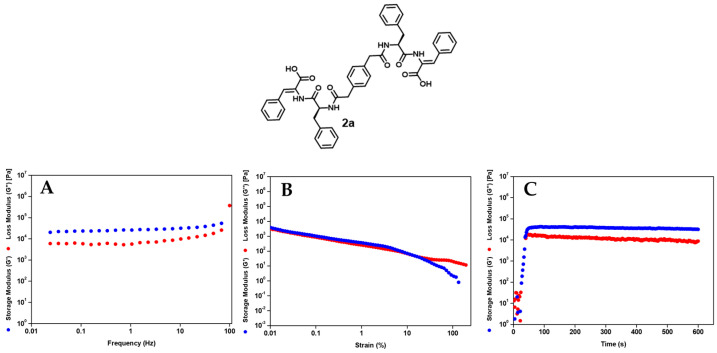
(**A**) Rheological protocol implemented for characterization of the bolaamphiphile hydrogels, illustrated for hydrogel **2a**: (**A**) frequency dependence of the shear elastic (G′) and loss (G″) moduli for hydrogel; (**B**) strain dependence of the shear elastic (G′) and loss (G″) moduli for hydrogel; (**C**) second kinetic process for gelation of hydrogel **2a** after mechanical breakup.

Frequency sweep experiments confirmed that in the studied frequency range, the storage modulus (G′) exceeded the loss modulus (G″) for all gelling compounds (**1c**, **1e**, and **2a**–**d**) (Table 3; Figure 6A) (Supporting Information, Figure S7) [17]. The relatively weak frequency dependence of G′ and the predominance of elastic over viscous response indicate that the mechanical integrity of the hydrogels results from reversible non-covalent physically cross-linked supramolecular networks, a hallmark of self-assembled peptide hydrogels.

Quantitative differences in mechanical strength are observed among the hydrogels. Hydrogels **1c**, **1e**, **2a** and **2b** display similar stiffness (G′ ~ 2.23–3.74 × 10^4^ Pa). Hydrogel **2d** displays the highest G′ (7.07 × 10^4^ Pa), while hydrogel **2c** exhibits a significantly lower elastic modulus (8.05 × 10^3^ Pa), qualifying as the weaker gel. This observation is in line with the network architecture of the hydrogels: while hydrogel **2c** shows a dense fibrillar structure made of short very thin fibers, hydrogel **2d** displays an entangled network made of longer and thicker fibers, suggesting a higher degree of fiber entanglement and stronger physical cross-links between the fibers. Network connectivity appears to be an important factor controlling the macroscopic stiffness of the hydrogels. [37]. Regarding aggregation efficacy (*CAC* values), no straightforward connection can be established between G′ and the CAC values. The strain sweep experiments provided further insight into the connection between gel stiffness, network robustness and yielding behavior (Figure 6B) (Supporting Information Figure S8). The strongest and the weakest hydrogels, **2d** and **2c**, respectively, undergo mechanical failure at widely different strain values, 4.26 and 144%, respectively, reflecting the architecture of their fibrillar networks. Hydrogel **2d** undergoes mechanical yielding at a low strain amplitude, consistent with a stiff, brittle hydrogel network formed by long and thick entangled fibers likely with strong cross-linking interactions (Figure 5F). In contrast, hydrogel **2c**, exhibiting a dense network of short, very thin fibers, probably weakly cross-linked, is able to sustain large deformations before breakdown (Figure 5E). The self-healing behavior of the bolaamphiphile hydrogels, relevant for injectable applications, were studied also by rheology (Figure 6C, Table 4) (Supporting Information, Figure S9).

Hydrogels **2a** and **2b** show full recovery after breakdown, while hydrogels **1e** and **2d** display partial recovery. Hydrogels **1c** and **2c** show negligeable recovery. The high extent of recovery seen for hydrogels **1e**, **2a**, **2b** and **2d** is likely related to their dense, highly interconnected fibrillar networks, which might allow a high density of reversible supramolecular cross-links capable of rapid reformation after shear. In contrast, hydrogels **1c** and **2c,** showing poor recovery, display high density of short thin fibers that probably do not support strong reversible physical cross-links.

Overall, the rheological data suggest that the macroscopic mechanical performance of the dehydropeptide-based bolaamphiphile hydrogels results from an intricate balance between aggregation propensity and network connectivity. Hydrogels **1e**, **2a**, **2b** and **2d**, exhibiting a range of elasticity values matching those of biological tissues, rapid gelation, and superb recovery, are well suited for applications that demand injectable properties [38].

#### 2.2.5. Biocompatibility and Drug Delivery Assays

**Figure 7:**
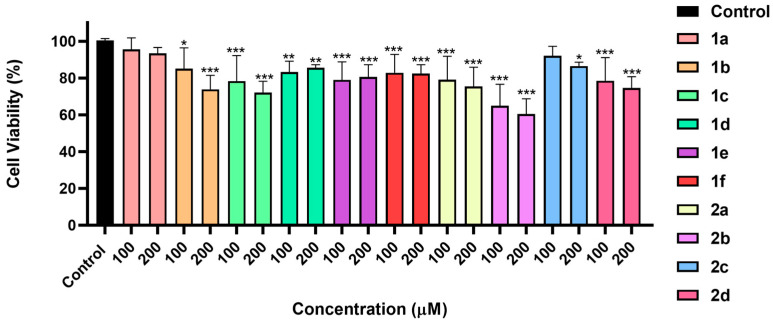
Viability of HaCaT cells treated with the dehydropeptide-based bolaamphiphiles for 24 h, at 100 and 200 µM concentration. * *p* < 0.05, ** *p* < 0.01, *** *p* < 0.001.

The cytocompatibility of the synthesized bolaamphiphilic dehydrodipeptides was evaluated using the MTT assay in HaCaT keratinocyte cells, a widely accepted in vitro model for preliminary assessment of biomaterials intended for biomedical and tissue-contact applications (Figure 7).

Overall, the majority of compounds exhibited good cytocompatibility, inducing only a modest reduction in cell viability (approximately 10–20%) at both tested concentrations, 100 and 200 μM. Importantly, the decrease in cell viability was essentially concentration-independent, suggesting that it is probably not associated with acute cytotoxicity but may instead result from transient cell–material interactions or mild perturbations of cellular metabolism.

**Figure 8:**
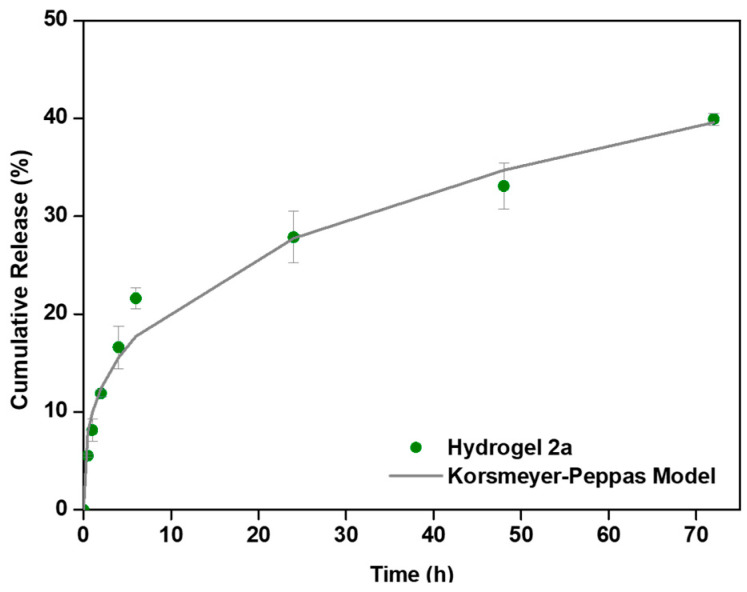
Release profile of MTX (0.3 mM) from hydrogel **2a** fitted to the Korsmeyer–Peppas Model.

The drug-release performance of the supramolecular hydrogels was evaluated using methotrexate (MTX, (2*S*)-2-[[4-[(2,4-diaminopteridin-6-yl)methyl-methylamino]benzoyl]amino]pentanedioic acid, C_20_H_22_N_8_O_5_, 454.44 g·mol^−1^) as a model anticancer agent. Compound **2a** was selected as a drug carrier due to its ability to form robust hydrogels at low critical gelation concentration (CGC), its elasticity being compatible with that of biological tissues, and its efficient recovery of mechanical properties following shear-induced disruption, which is essential for injectable drug delivery systems. The thermal stability of hydrogel **2a** was further assessed, revealing a gel–sol transition temperature of 95 °C (Supporting Information, Figure S10). This relatively high transition temperature indicates the formation of a highly stable supramolecular network. From a biomedical perspective, this elevated gel–sol transition temperature is particularly advantageous, as it ensures that the hydrogel remains structurally intact under physiological conditions (≈37 °C) and during handling and administration. Moreover, the large thermal gap between body temperature and the transition temperature suggests a significant resistance to premature gel disassembly, which is critical for maintaining sustained drug release and structural integrity in vivo. MTX-loaded hydrogels (0.3 mM) were prepared by incorporating the drug during gel formation. Drug-release profiles were obtained under physiological conditions (PBS, pH 7.4) by monitoring drug release over time (Figure 8). Hydrogel **2a** exhibited sustained MTX release, with approximately 40% of the incorporated MTX released over 72 h, suggesting effective retention of the drug within the supramolecular network, likely due to a combination of mesh-size-restricted drug diffusion and non-covalent interactions between MTX and the peptide-based fibrillar matrix. Such partial release over several days is desirable for localized therapies, where prolonged drug availability is required.

To get insight into the release mechanism of MTX from hydrogel **2a**, the experimental data were fitted to the Korsmeyer–Peppas [39], Peppas–Sahlin [40], and Weibull models [41] (Table 5) (Supporting Information, Figure S10).

The Korsmeyer–Peppas model afforded better fitting (*R*^2^ value) of the experimental cumulative drug-release data than the Peppas–Sahlin [40] and Weibull models [41] (Supporting Information, Table S1). The MTX diffusion exponent *n* ~ 0.32 from hydrogel **2a** indicates a Fickian diffusion-controlled release mechanism, consistent with a stable, dynamic supramolecular network that displays sufficient stiffness to retain the drug while allowing controlled diffusion through the hydrated matrix.

Overall, these results demonstrate that hydrogel **2a** combines mechanical robustness, injectability and sustained, diffusion-controlled drug release, highlighting its potential as a versatile platform for minimally invasive, localized drug delivery applications.

## 3. Conclusions

This study establishes symmetrical bolaamphiphilic dehydrodipeptides as a versatile platform for injectable supramolecular hydrogels with tunable structure–property–function relationships. Systematic variation in the dehydroamino acid residue and linker architecture revealed that efficient aggregation, gelation, and mechanical robustness arise from a balance between hydrophobicity, aromatic interactions, and packing efficiency rather than hydrophobicity alone. Aggregation and gelation studies, combined with TEM and rheology, demonstrated that network connectivity at the nanoscale directly governs macroscopic mechanical behavior and self-healing capacity, defining rheological profiles suitable for injectability. The hydrogels exhibited good cytocompatibility, and a representative system (compound **2a**) showed sustained, diffusion-controlled release of methotrexate under physiological conditions. Collectively, these findings provide clear design principles for the development of peptide-based supramolecular hydrogels as functional biomaterials for minimally invasive drug delivery.

## 4. Materials and Methods

### 4.1. Synthesis

Chemicals, analytical grade reagents and solvents were acquired from Acros (Gell, Belgium) and Sigma-Aldrich (St. Louis, MO, USA). Bolaamphiphiles were synthesized using methods developed in our laboratory and characterized by ^1^H and ^13^C NMR spectrometry (NMR) and High-Resolution Mass Spectrometry (HRM). NMR spectra were recorded on a Bruker Avance III 400 spectrometer (Billerica, MA, USA) at 400.13 MHz for ^1^H and 100.62 MHz ^13^C. The NMR spectra was recorded at 25 °C. Deuterated dimethyl sulfoxide (DMSO-d_6_) was used as solvent. Chemical shifts are given in parts per million (ppm) and the coupling constants in Hertz (Hz). HRMS data were recorded by the mass spectrometry service of the University of Vigo, Spain. MS was recorded by a Thermo Finnigan LxQ (Linear Ion Trap) Mass Detector with Electro Spray Ionization (ESI) (Linear Ion Trap, San Jose, CA, USA).

#### 4.1.1. Synthesis of Boc-L-Phe-D,L-Thr-OMe (**3**)

Boc-L-Phe-OH (1.0 equiv., 6.10 mmol, 1.60 g) was dissolved in MeCN (50 mL) and cooled to 0 °C. HBTU (1.1 equiv., 6.71 mmol, 2.54 g), DL-Thr-OMe (1.0 equiv., 6.10 mmol, 1.00 g), Et_3_N (3.0 equiv., 18.3 mmol, 2.52 mL) were added sequentially and the mixture was stirred at rt overnight. The solvent was removed under reduced pressure to afford a residue that was partitioned between EtOAc (50 mL) and KHSO_4_ (1 M, 50 mL). After separation of the phases, the organic phase was thoroughly washed with KHSO_4_ (1 M, 2 × 50 mL), NaHCO_3_ (1 M, 3 × 50 mL), and brine (3 × 50 mL) and then dried with anhydrous MgSO_4_. Filtration followed by removal of the solvent under reduced pressure afforded compound **3** as a diasteroisomeric mixture with a 50% yield. (1.16 g, 3.05 mmol). ^1^H NMR (400 MHz, DMSO-d_6_, δ): 1.02 (3H, dd, *J* = 6.4 and 32.8 Hz, CH_3_), 1.29 (9H, s, O_2_C(CH_3_)_3_ Boc), 2.73–2.75 (1H, m, β-CH_2_ Phe), 2.98–3.03 (1H, m, β-CH_2_ Phe), 3.63 (3H, s, OCH_3_), 4.10–4.12 (1H, m, α-CH Thr), 4.28–4.31 (1H, m, α-CH (β-OH)), 5.03–5.06 (1H, m, β-CH (β-OH)), 7.03 (1H, dd, *J* = 8.8 and 25.2 Hz, NH), 7.15–7.29 (5H, m, ArH Phe), 7.88 (1H, dd, *J* = 8.8 and 25.6 Hz, NH) ppm.

#### 4.1.2. Synthesis of Boc-L-Phe-Z-ΔThr-OMe (**4**)

Compound **3** (1.0 equiv., 1.16 g, 3.05 mmol) was dissolved in dry MeCN (5 mL). Boc_2_O (1.20 equiv., 1.09 mg, 5.00 mmol) and DMAP (0.12 equiv., 0.061 mg, 0.5 mmol) were added. The mixture was stirred with a calcium chloride tube. The reaction was monitored using ^1^H NMR. After the disappearance of the starting material was observed, TMG (2% v/v, 0.1 mL) was added. Once all the intermediates had reacted, the solvent was removed, and the residue was dissolved in ethyl acetate (50 mL). The organic phase was washed with KHSO_4_ 1 M, NaHCO_3_ 1 M and brine (3 × 30 mL each). The organic phase was dried with anhydrous magnesium sulphate, and the solvent was removed under reduced pressure. Compound **4** was obtained in 83% yield. (0.779 g, 2.40 mmol). ^1^H NMR (400 MHz, DMSO-d_6_, δ): 1.30 (9H, s, O_2_C(CH_3_)_3_ Boc), 1.62 (3H, d, J = 6.8 Hz, CH_3_), 2.75–2.81 (1H, m, β-CH_2_ Phe), 2.98–3.02 (1H, m, β-CH_2_ Phe), 3.64 (3H, s, OCH_3_), 4.26–4.31 (1H, m, α-CH Phe), 6.53 (1H, q, J = 6.8 Hz, β-CH ΔAbu), 6.96 (1H, d, J = 8.4 Hz, NH), 7.18–7.31 (5H, m, ArH Phe), 9.29 (1H, s, NHΔAbu) ppm. ^13^C NMR (100.6 MHz, DMSO-d_6_, δ): 13.40 (CH_3_, CH_3_ ΔAbu), 28.09 (3 x CH_3_, OC(CH_3_)_3_), 37.28 (CH_2_, β-CH_2_ Phe), 51.80 (CH_3_, OCH_3_), 55.69 (CH, α-CH Phe), 77.98 (C, OC(CH_3_)_3_), 126.18 (CH, CH Phe), 127.61 (C), 127.99 (CH, CH Phe), 129.22 (CH, CH Phe), 132.46 (CH, CH ΔAbu), 138.03 (C), 155.26 (C, C=O Boc), 164.57 (C), 170.85 (C, C=O Phe) ppm.

#### 4.1.3. Synthesis of Compound (**5a**)

Compound **4** (2.2 equiv., 1.39 mmol, 526 mg) was dissolved in TFA (2 mL) during 1 h. Ethyl ether was added, and the solvent was evaporated under reduced pressure. Then, we added DMF (5 mL), naphthalene-2,6-dicarboxylic acid 1.0 equiv., 0.63 mmol, 136 mg), HBTU (2.4 equiv., 1.50 mmol, 575 mg) and Et_3_N (6.0 equiv., 3.80 mmol, 0.53 mL). The reaction was left stirring at room temperature overnight. Water (25 mL) was added to the reaction to precipitate. The solid was filtrated to afford compound **5a** (270 mg, 0.38 mmol, 60%), as a brown solid. 1H-NMR (400 MHz, DMSO-d_6_, δ): 1.68 (6H, d, *J* = 6.8 Hz, CH_3_ ΔAbu), 3.06–3.12 (2H, dd, *J* = 10.8 and 13.6 Hz, β-CH_2_ Phe), 3.20–3.24 (2H, dd, *J* = 4.4 and 14. Hz, β-CH_2_ Phe), 3.65 (6H, s, OCH_3_), 4.90–4.96 (2H, m, α-CH Phe), 6.55–6.61 (2H, q, *J* = 6.8 and 7.2 Hz, β-CH ΔAbu), 7.15–7.18 (2H, t, *J* = 7.2 Hz, ArH_4′_ Phe), 7.24–7.29 (4H, m, ArH_3′_ Phe), 7.41–7.43 (4H, m, ArH_2′_ Phe), 7.92 (2H, d, *J* = 8.40 Hz, ArH_3_ central ring), 8.04 (2H, d, *J* = 8.80 Hz, ArH_4_ central ring), 8.44 (2H, s, ArH_1_ central ring), 8.87 (2H, d, *J* = 8.4 Hz, NH Phe), 9.54 (2H, s, NH ΔAbu) ppm. ^13^C-NMR (100.6 MHz, DMSO-d_6_ δ): 13.5 (CH_3_, CH_3_ ΔAbu), 37.2 (CH_2_, β-CH_2_ Phe), 51.9 (CH_3_, OCH_3_), 54.9 (CH, α-CH Phe), 64.9 (C), 125.0 (CH, C_3_H central ring), 126.3 (CH, ArC_4′_H Phe), 127.5 (CH, C_1_H central ring), 127.6 (C), 128.1 (CH, ArC_3′_H Phe), 128.8 (CH, C_4_H central ring), 129.3 (CH, ArC_2′_H Phe), 132.8 (CH, β-CH ΔAbu), 133.2 (C), 138.2 (C), 164.6 (C, C=O ΔAbu), 166.1 (C, C=O central ring), 170.6 (C, C=O Phe) ppm.

#### 4.1.4. Synthesis of Compound (**5b**)

Compound **4** (2.4 equiv., 1.3 mmol, 500 mg) was dissolved in TFA (2 mL) during 1 h. Ethyl ether was added, and the solvent was evaporated under reduced pressure. Then, we added DMF (5 mL), terephthalic acid (1.0 equiv., 0.554 mmol, 92 mg), HBTU (2.4 equiv., 1.3 mmol, 504 mg) and Et_3_N (6.0 equiv., 3.32 mmol, 0.463 mL). The reaction was left stirring at room temperature overnight. Water (25 mL) was added to the reaction to precipitate. The solid was filtrated to afford compound **5b** (264 mg, 0.403 mmol, 73%), as a white solid. ^1^H-NMR (400 MHz, DMSO-d_6_, δ): 1.67 (6H, d, *J* = 7.2 Hz, CH_3_ ΔAbu), 3.00–3.07 (2H, app.t, β-CH_2_ Phe), 3.16–3.21 (2H, dd, *J* = 4.00 and 13.6 Hz, β-CH_2_ Phe), 3.64 (6H, s, OCH_3_), 4.82–4.89 (2H, m, α-CH Phe), 6.54–6.60 (2H, q, *J* = 7.20 and 14.0 Hz, β-CH ΔAbu), 7.14–7.18 (2H, t, *J* = 7.6 Hz, ArH Phe), 7.24–7.28 (4H, t, *J* = 7.6 Hz, ArH Phe), 7.39 (4H, d, *J* = 7.2 Hz, ArH Phe), 7.83 (4H, s, ArH central ring), 8.75 (2H, d, *J* = 8.4 Hz, NH Phe), 9.50 (2H, s, NH ΔAbu) ppm. ^13^C-NMR (100.6 MHz, DMSO-d_6_ δ): 13.5 (CH_3_, CH_3_ ΔAbu), 37.1 (CH_2_, β-CH_2_ Phe), 51.9 (CH_3_, OCH_3_), 54.8 (CH, α-CH Phe), 126.3 (CH, ArCH Phe), 127.3 (CH, ArCH central ring), 127.6 (C), 128.1 (CH, ArCH Phe), 129.2 (CH, ArCH Phe), 132.7 (CH, β-CH ∆Abu), 136.3 (C), 138.2 (C), 164.5 (C, C=O Phe), 165.6 (C, C=O central ring), 170.5 (C, C=O ∆Abu) ppm.

#### 4.1.5. Synthesis of Compound (**5c**)

Compound **4** (2.2 equiv., 1.10 mmol, 400 mg) was dissolved in TFA (2 mL) during 1 h. Ethyl ether was added, and the solvent was evaporated under reduced pressure. Then, we added DMF (5 mL), 2,2′-(1,4-Phenylene)diacetic acid (1.0 equiv., 0.503 mmol, 97 mg), HBTU (2.2 equiv., 0.110 mmol, 417 mg) and Et_3_N (6.0 equiv., 3.01 mmol, 0.420 mL). The reaction was left stirring at room temperature overnight. Water (25 mL) was added to the reaction to precipitate. The solid was filtrated to afford compound **5c** (200 mg, 0.300 mmol, 60%), as a white solid. ^1^H-NMR (400 MHz, DMSO-d_6_, δ): 1.58 (6H, d, *J* = 7.2 Hz, CH_3_ ΔAbu), 2.72–2.83 (2H, m, β-CH_2_ Phe), 3.03–3.10 (2H, m, β-CH_2_ Phe), 3.29–3.32 (4H, m, CH_2_ central ring), 3.61 (6H, s, OCH_3_), 4.64–4.70 (2H, m, α-CH Phe), 6.50–6.55 (2H, q, *J* = 6.8 and 7.2 Hz, β-CH ΔAbu), 6.92 (4H, s, ArH central ring), 7.17–7.26 (10H, m, ArH Phe), 8.33 (2H, d, *J* = 8.4 Hz, NH Phe), 9.40 (2H, s, NH ΔAbu) ppm. ^13^C-NMR (100.6 MHz, DMSO-d_6_ δ): 13.4 (CH_3_, CH_3_ ΔAbu), 37.8 (CH_2_, β-CH_2_ Phe), 41.6 (CH_2_, central ring), 51.8 (CH_3_, OCH_3_), 53.8 (CH, α -CH Phe), 126.3 (CH, ArCH Phe), 127.6 (C), 127.9 (CH, ArCH Phe), 128.6 (CH, ArCH central ring), 129.2 (CH, ArCH Phe), 132.5 (CH, β-CH ∆Abu), 134.1 (C), 137.6 (C), 164.5 (C, C=O), 170.0 (C, C=O), 170.1 (C, C=O) ppm.

#### 4.1.6. Synthesis of Compound (**5d**)

Compound **4** (2.2 equiv., 1.90 mmol, 700 mg) was dissolved in TFA (2 mL) during 1 h. Ethyl ether was added, and the solvent was evaporated under reduced pressure. Then, we added DMF (5 mL), succinc acid (1.0 equiv., 0.860 mmol, 102 mg), HBTU (2.2 equiv., 1.90 mmol, 720 mg) and Et_3_N (6.0 equiv., 5.16 mmol, 0.720 mL). The reaction was left stirring at room temperature overnight. Water (25 mL) was added to the reaction to precipitate. The solid was filtrated to afford compound **5d** (253 mg, 0.420 mmol, 50%), as a white solid. ^1^H-NMR (400 MHz, DMSO-d_6_, δ): 1.59 (6H, d, *J* = 7.2 Hz, CH_3_ ΔAbu), 2.22–2.29 (4H, m, CH_2_ succinic), 2.74–2.79 (2H, m, β-CH_2_ Phe), 3.03–3.08 (2H, m, β-CH_2_ Phe), 3.63 (6H, s, OCH_3_), 4.55–4.61 (2H, m, α-CH Phe), 6.50–6.55 (2H, q, *J* = 7.2 Hz, β-CH ΔAbu), 7.12–7.26 (10H, m, ArH Phe), 8.19 (2H, d, *J* = 8.4 Hz, NH Phe), 9.31 (2H, s, NH ΔAbu) ppm. ^13^C-NMR (100.6 MHz, DMSO-d_6_ δ): 13.3 (CH_3_, CH_3_ ΔAbu), 30.6 (CH_2_, CH_2_ succinic), 37.5 (CH_2_, β-CH_2_ Phe), 51.8 (CH_3_, OCH_3_), 53.9 (CH, α-CH Phe), 126.3 (CH, ArCH Phe), 127.7 (C, C ΔAbu), 128.0 (CH, ArCH Phe), 129.1 (CH, ArCH Phe), 132.6 (CH, β-CH ΔAbu), 137.8 (C, C Phe), 164.5 (C, C=O ΔAbu), 170.4 (C, C=O Phe), 171.5 (C, C=O Succinic) ppm.

#### 4.1.7. Synthesis of Compound (**5e**)

Compound **4** (2.4 equiv., 1.60 mmol, 600 mg) was dissolved in TFA (2 mL) during 1 h. Ethyl ether was added, and the solvent was evaporated under reduced pressure. Then, we added DMF (5 mL), suberic acid (1.0 equiv., 0.664 mmol, 116 mg), HBTU (2.4 equiv., 1.59 mmol, 604 mg) and Et_3_N (6.0 equiv., 3.98 mmol, 0.55 mL). The reaction was left stirring at room temperature overnight. Water (25 mL) was added to the reaction to precipitate. The solid was filtrated to afford compound **5e** (307 mg, 0.463 mmol, 70%), as a white solid. ^1^H-NMR (400 MHz, DMSO-d_6_, δ): 0.94–0.99 (4H, m, CH_2_ suberic), 1.22–1.29 (4H, m, CH_2_ suberic), 1.60 (6H, d, *J* = 7.2 Hz, CH_3_ ΔAbu), 1.94–2.00 (4H, m, CH_2_ suberic), 2.74–2.80 (2H, m, β-CH_2_ Phe), 3.02–3.09 (2H, m, β-CH_2_ Phe), 3.63 (6H, s, OCH_3_), 4.64–4.70 (2H, m, α-CH Phe), 6.50–6.55 (2H, q, *J* = 6.8 and 7.2 Hz, β-CH ΔAbu), 7.14–7.29 (10H, m, ArH Phe), 8.04 (2H, d, *J* = 8.8 Hz, NH Phe), 9.35 (2H, s, NH ΔAbu) ppm. ^13^C-NMR (100.6 MHz, DMSO-d_6_ δ): 13.4 (CH_3_, CH_3_ ΔAbu), 25.1 (CH_2_, CH_2_ suberic), 28.2 (CH_2_, CH_2_ suberic), 35.1 (CH_2_, CH_2_ suberic), 37.7 (CH_2_, β-CH_2_ Phe), 51.9 (CH_3_, OCH_3_), 53.6 (CH, α-CH Phe), 126.2 (CH, ArCH Phe), 127.6 (C, C ΔAbu), 128.0 (CH, ArCH Phe), 129.2 (CH, ArCH Phe), 132.6 (CH, β-CH ΔAbu), 137.9 (C, C Phe), 164.6 (C, C=O ΔAbu), 170.7 (C, C=O Phe), 172.1 (C, C=O Suberic) ppm.

#### 4.1.8. Synthesis of Compound (**5f**)

Compound **4** (2.2 equiv., 0.990 mmol, 360 mg) was dissolved in TFA (2 mL) during 1 h. Ethyl ether was added, and the solvent was evaporated under reduced pressure. Then, we added DMF (5 mL), azelaic acid (1.0 equiv., 0.450 mmol, 85 mg), HBTU (2.4 equiv., 1.80 mmol, 410 mg) and Et_3_N (6.0 equiv., 2.70 mmol, 0.376 mL). The reaction was left stirring at room temperature overnight. Water (25 mL) was added to the reaction to precipitate. The solid was filtrated to afford compound **5f** (208 mg, 0.307 mmol, 68%), as a white solid. ^1^H-NMR (400 MHz, DMSO-d_6_, δ): 0.94–1.06 (6H, m, C*H*_2_ azelaic), 1.29–1.33 (4H, m, C*H*_2_ azelaic), 1.60 (6H, d, *J* = 6.8 Hz, C*H*_3_ ΔAbu), 2.00 (4H, t, *J* = 7.2 Hz, C*H*_2_ azelaic), 2.73–2.79 (2H, m, β-C*H*_2_ Phe), 3.02–3.07 (2H, m, β-C*H*_2_ Phe), 3.63 (6H, s, OC*H*_3_), 4.63–4.69 (2H, m, α-C*H* Phe), 6.50–6.55 (2H, q, *J* = 7.2 Hz, β-C*H* ΔAbu), 7.15–7.28 (10H, m, Ar*H* Phe), 8.04 (2H, d, *J* = 8.4Hz, N*H* Phe), 9.34 (2H, s, N*H* ΔAbu) ppm. ^13^C-NMR (100.6 MHz, DMSO-d_6_ δ): 13.5 (CH_3_, *C*H_3_ ΔAbu), 25.1 (CH_2_, *C*H_2_ azelaic), 28.4 (CH_2_, *C*H_2_ azelaic), 28.7 (CH_2_, *C*H_2_ azelaic), 35.3 (CH_2_, *C*H_2_ azelaic), 37.9 (CH_2_, β-*C*H_2_ Phe), 52.1 (CH_3_, O*C*H_3_), 54.1 (CH, α-*C*H Phe), 127.1 (CH, Ar*C*H Phe), 128.5 (C), 128.2 (CH, Ar*C*H Phe), 129.5 (CH, Ar*C*H Phe), 132.5 (CH, β-*C*H ΔAbu), 138.1 (C), 166.4 (C, *C*=O), 170.5 (C, *C*=O), 172.5 (C, *C*=O) ppm.

#### 4.1.9. Synthesis of Compound (**1a**)

Compound **5a** (0.35 mmol, 250 mg) was dissolved in 1,4-dioxane (5 mL) and NaOH (1 M) (6.00 equiv., 2.1 mL) was added to the solution. The reaction was monitored by TLC. When all the starting material was consumed, the organic solvent was removed under reduced pressure, and the reaction mixture was acidified to pH 3 with KHSO_4_ (1 M). The solid precipitate was filtered to afford bolaamphiphile **1a** (205 mg, 0.301 mmol, 85%), as a pink solid. ^1^H-NMR (400 MHz, DMSO-d_6_, δ): 1.65 (6H, d, *J* = 7.2 Hz, C*H*_3_ ΔAbu), 3.05–3.11 (2H, t app., β-C*H*_2_ Phe), 3.20–3.25 (2H, dd, *J* = 3.6 and 13.6 Hz, β-C*H*_2_ Phe), 4.91–4.96 (2H, m, α-C*H* Phe), 6.55–6.62 (2H, q, *J* = 6.80 and 7.20 Hz, β-C*H* ΔAbu), 7.15 (2H, t, *J* = 7.2 Hz, Ar*H*_4′_ Phe), 7.26 (4H, t, *J* = 8.0 Hz, Ar*H*_3′_ Phe), 7.43 (4H, d, *J* = 7.6 Hz, Ar*H*_2′_ Phe), 7.92 (2H, d, *J* = 8.4 Hz, Ar*H*_3_ central ring), 8.04 (2H, d, *J* = 8.8 Hz, Ar*H*_4_ central ring), 8.43 (2H, s, Ar*H*_1_ central ring), 8.84 (2H, d, *J* = 8.4 Hz, N*H* Phe), 9.38 (2H, s, N*H* ΔAbu), 12.4 (2H, s, CO_2_*H*) ppm. ^13^C-NMR (100.6 MHz, DMSO-d_6_ δ): 13.8 (CH_3_, *C*H_3_ ΔAbu), 37.2 (CH_2_, β-*C*H_2_ Phe), 55.0 (CH, α-*C*H Phe), 125.0 (CH, *C*_3_H central ring), 126.3 (CH, Ar*C*_4′_H Phe), 127.5 (CH, *C*_1_H central ring), 128.1 (CH, Ar*C*_3′_H Phe), 128.2 (C), 128.8 (CH, *C*_4_H central ring), 129.2 (CH, Ar*C*_2′_H Phe), 132.3 (CH, β-*C*H ΔAbu), 132.8 (C), 133.2 (C), 138.3 (C), 165.5 (C, *C*=O ΔAbu), 166.1 (C, *C*=O central ring), 170.2 (C, *C*=O Phe) ppm. HRMS (ESI): [M+H] calcd. for C_38_H_37_N_4_O_8_ 677.2611; found 677.2632.

#### 4.1.10. Synthesis of Compound (**1b**)

Compound **5b** (0.394 mmol, 258mg) was dissolved in 1,4-dioxane (5 mL) and NaOH (1 M) (6.00 equiv., 2.36 mL). The reaction was monitored by TLC. When all the starting material was consumed, the organic solvent was removed under reduced pressure, and the reaction mixture was acidified to pH 3 with KHSO_4_ (1 M). The solid precipitate was filtered to afford bolaamphiphile **1b** (222 mg, 0.354 mmol, 90%), as a white solid. ^1^H-NMR (400 MHz, DMSO-d_6_, δ): 1.64 (6H, d, *J* = 7.2 Hz, C*H*_3_ ΔAbu), 3.00–3.06 (2H, app.t, β-C*H*_2_ Phe), 3.17–3.21 (2H, dd, *J* = 3.6 and 13.6 Hz, β-C*H*_2_ Phe), 4.84–4.90 (2H, m, α-C*H* Phe), 6.56–6.61 (2H, q, *J* = 7.2 and 14.4 Hz, β-C*H* ΔAbu), 7.13–7.17 (2H, app t, Ar*H* Phe), 7.23–7.27 (4H, app t, Ar*H* Phe), 7.39 (4H, d, *J* = 8.0 Hz, Ar*H* Phe), 7.83 (4H, s, Ar*H* central ring), 8.72 (2H, d, *J* = 8.8 Hz, N*H* Phe), 9.38 (2H, s, N*H* ΔAbu), 12.4 (2H, s, CO_2_*H*) ppm. ^13^C-NMR (100.6 MHz, DMSO-d_6_ δ): 13.7 (CH_3_, *C*H_3_ ΔAbu), 37.3 (CH_2_, β-*C*H_2_ Phe), 54.9 (CH, α -*C*H Phe), 126.3 (CH, Ar*C*H Phe), 127.3 (CH, Ar*C*H central ring), 128.0 (CH, Ar*C*H Phe), 128.2 (C), 129.2 (CH, Ar*C*H Phe), 132.5 (CH, β-*C*H ∆Abu), 136.3 (C), 138.3 (C), 165.5 (C, *C*=O), 165.6 (C, *C*=O), 170.1 (C, *C*=O) ppm. HRMS (ESI): [M+H] calcd. for C_34_H_35_N_4_O_8_ 627.2455; found 677.2501.

#### 4.1.11. Synthesis of Compound (**1c**)

Compound **5c** (0.300 mmol, 200 mg) was dissolved in 1,4-dioxane (10 mL) and NaOH (1 M) (6.00 equiv., 1.80 mL). The reaction was monitored by TLC. When all the starting material was consumed, the organic solvent was removed under reduced pressure, and the reaction mixture was acidified to pH 3 with KHSO_4_ (1 M). The solid precipitate was filtered to afford compound **1c** (135 mg, 0.210 mmol, 70%), as a white solid. ^1^H-NMR (400 MHz, DMSO-d_6_, δ): 1.56 (6H, d, *J* = 7.2 Hz, C*H*_3_ ΔAbu), 2.75–2.82 (2H, m, β-C*H*_2_ Phe), 3.04–3.09 (2H, dd, *J* = 4.8 and 14 Hz, β-C*H*_2_ Phe), 3.28–3.37 (4H, q, *J* = 14 Hz, C*H*_2_ central ring), 4.64–4.70 (2H, m, α-C*H* Phe), 6.52–6.58 (2H, q, *J* = 6.8 and 7.2 Hz, β-C*H* ΔAbu), 6.89 (4H, s, Ar*H* central ring), 7.15–7.26 (10H, m, Ar*H* Phe), 8.30 (2H, d, *J* = 8.4 Hz, N*H* Phe), 9.25 (2H, s, N*H* ΔAbu) ppm. ^13^C-NMR (100.6 MHz, DMSO-d_6_ δ): 13.6 (CH_3_, *C*H_3_ ΔAbu), 37.9 (CH_2_, β-*C*H_2_ Phe), 41.6 (CH_2_, central ring), 53.8 (CH, α -*C*H Phe), 126.3 (CH, Ar*C*H Phe), 128.0 (CH, Ar*C*H Phe), 128.1 (C), 128.6 (CH, Ar*C*H central ring), 129.3 (CH, Ar*C*H Phe), 132.3 (CH, β-*C*H ∆Abu), 134.1 (C), 137.7 (C), 165.5 (C, *C*=O), 170.0 (C, *C*=O), 170.1 (C, *C*=O) ppm. HRMS (ESI): [M+H] calcd. for C_36_H_39_N_4_O_8_ 655.2768; found 655.2772.

#### 4.1.12. Synthesis of Compound (**1d**)

Compound **5d** (0.420 mmol, 253 mg) was dissolved in 1,4-dioxane (6.0 mL) and NaOH (1 M) (6.00 equiv., 2.52 mmol, 2.52 mL). The reaction was monitored by TLC. When all the starting material was consumed, the organic solvent was removed under reduced pressure, and the reaction mixture was acidified to pH 3 with KHSO_4_ (1 M). The solid precipitate was filtered to afford bolaamphiphile **1d** (195 mg, 0.330 mmol, 78%), as a white solid. ^1^H-NMR (400 MHz, DMSO-d_6_, δ): 1.56 (6H, d, *J* = 6.8 Hz, C*H*_3_ ΔAbu), 2.11–2.15 (4H, m, C*H*_2_ succinic), 2.72–2.78 (2H, m, β-C*H*_2_ Phe), 3.04–3.08 (2H, dd, *J* = 4 and 14 Hz, β-C*H*_2_ Phe), 4.56–4.62 (2H, m, α-C*H* Phe), 6.51–6.57 (2H, q, *J* = 7.2 Hz, β-C*H* ΔAbu), 7.15–7.27 (10H, m, Ar*H* Phe), 8.14 (2H, d, *J* = 8.4 Hz, N*H* Phe), 9.17 (2H, s, N*H* ΔAbu), 12.4 (2H, s, CO_2_*H*) ppm. ^13^C-NMR (100.6 MHz, DMSO-d_6_ δ): 13.6 (CH_3_, *C*H_3_ ΔAbu), 30.6 (CH_2_, *C*H_2_ succinic), 37.6 (CH_2_, β-*C*H_2_ Phe), 53.9 (CH, α-*C*H Phe), 126.2 (CH, Ar*C*H Phe), 128.0 (CH, Ar*C*H Phe), 128.2 (C, *C* ΔAbu), 129.2 (CH, Ar*C*H Phe), 132.2 (CH, β-*C*H ΔAbu), 138.0 (C, *C* Phe), 165.4 (C, *C*=O ΔAbu), 170.4 (C, *C*=O Phe), 171.4 (C, *C*=O Succinic) ppm. HRMS (ESI): [M+H] calcd. for C_30_H_35_N_4_O_8_ 579.2455; found 579.2448.

#### 4.1.13. Synthesis of Compound (**1e**)

Compound **5e** (0.463 mmol, 307 mg) was dissolved in 1,4-dioxane (6 mL) and NaOH (1 M) (6.00 equiv., 2.78 mL). The reaction was monitored by TLC. When all the starting material was consumed, the organic solvent was removed under reduced pressure, and the reaction mixture was acidified to pH 3 with KHSO_4_ (1 M). The solid precipitate was filtered to afford bolaamphiphile **1e** (200 mg, 0.315 mmol, 70%), as a white solid. ^1^H-NMR (400 MHz, DMSO-d_6_, δ): 0.94–1.04 (4H, m, C*H*_2_ suberic), 1.22–1.33 (4H, m, C*H*_2_ suberic), 1.58 (6H, d, *J* = 6.8 Hz, C*H*_3_ ΔAbu), 1.98 (4H, t, *J* = 7.2 Hz, C*H*_2_ suberic), 2.73–2.79 (2H, m, β-C*H*_2_ Phe), 3.03–3.07 (2H, dd, *J* = 4 and 13.6 Hz, β-C*H*_2_ Phe), 4.64–4.70 (2H, m, α-C*H* Phe), 6.52–6.58 (2H, q, *J* = 7.2 Hz, β-C*H* ΔAbu), 7.15–7.29 (10H, m, Ar*H* Phe), 8.01 (2H, d, *J* = 8.4 Hz, N*H* Phe), 9.20 (2H, s, N*H* ΔAbu), 12.4 (2H, s, CO_2_*H*) ppm. ^13^C-NMR (100.6 MHz, DMSO-d_6_ δ): 13.6 (CH_3_, *C*H_3_ ΔAbu), 25.0 (CH_2_, *C*H_2_ suberic), 28.2 (CH_2_, *C*H_2_ suberic), 35.2 (CH_2_, *C*H_2_ suberic), 37.8 (CH_2_, β-*C*H_2_ Phe), 53.7 (CH, α-*C*H Phe), 126.2 (CH, Ar*C*H Phe), 128.0 (CH, Ar*C*H Phe), 128.1 (C, *C* ΔAbu), 129.0 (CH, Ar*C*H Phe), 132.2 (CH, β-*C*H ΔAbu), 137.9 (C, *C* Phe), 165.5 (C, *C*=O ΔAbu), 170.3 (C, *C*=O Phe), 172.1 (C, *C*=O Suberic) ppm. HRMS (ESI): [M+H] calcd. for C_34_H_43_N_4_O_8_ 635.3081; found 6635.3102.

#### 4.1.14. Synthesis of Compound (**1f**)

Compound **5f** (0.807 mmol, 208 mg) was dissolved in 1,4-dioxane (5.0 mL) and NaOH (1 M) (6.00 equiv., 1.84 mmol, 1.84 mL). The reaction was monitored by TLC. When all the starting material was consumed, the organic solvent was removed under reduced pressure, and the reaction mixture was acidified to pH 3 with KHSO_4_ (1 M). The solid precipitate was filtered to afford bolaamphiphile **1f** (180 mg, 0.277 mmol, 89%), as a white solid. ^1^H-NMR (400 MHz, DMSO-d_6_, δ): 0.94–1.06 (6H, m, C*H*_2_ azelaic), 1.27–1.33 (4H, m, C*H*_2_ azelaic), 1.58 (6H, d, *J* = 7.2 Hz, C*H*_3_ ΔAbu), 1.99 (4H, t, *J* = 7.6 Hz, C*H*_2_ azelaic), 2.73–2.80 (2H, m, β-C*H*_2_ Phe), 3.03–3.07 (2H, dd, *J* = 4.4 and 13.6 Hz, β-C*H*_2_ Phe), 4.64–4.70 (2H, m, α-C*H* Phe), 6.52–6.58 (2H, q, *J* = 7.2 Hz, β-C*H* ΔAbu), 7.14–7.29 (10H, m, Ar*H* Phe), 8.02 (2H, d, *J* = 8.8 Hz, N*H* Phe), 9.19 (2H, s, N*H* ΔAbu) ppm. ^13^C-NMR (100.6 MHz, DMSO-d_6_ δ): 13.6 (CH_3_, *C*H_3_ ΔAbu), 25.1 (CH_2_, *C*H_2_ azelaic), 28.3 (CH_2_, *C*H_2_ azelaic), 28.6 (CH_2_, *C*H_2_ azelaic), 35.2 (CH_2_, *C*H_2_ azelaic), 37.8 (CH_2_, β-*C*H_2_ Phe), 53.7 (CH, α-*C*H Phe), 126.2 (CH, Ar*C*H Phe), 128.1 (C), 127.9 (CH, Ar*C*H Phe), 129.2 (CH, Ar*C*H Phe), 132.2 (CH, β-*C*H ΔAbu), 137.9 (C), 165.5 (C, *C*=O), 170.3 (C, *C*=O), 172.1 (C, *C*=O) ppm. HRMS (ESI): [M+H] calcd. for C_35_H_45_N_4_O_8_ 649.3227; found 649.3234.

#### 4.1.15. Synthesis of Boc-L-Phe-D,L-Phe(β-OH)-OMe (**6**)

Boc-L-Phe-OH (1.0 equiv., 4.00 mmol, 1.10 g) was dissolved in MeCN (40 mL) and cooled to 0 °C. H-D,L-Phe(β-OH)-OMe (1.0 equiv., 4.00 mmol, 0.93 g), Et_3_N (3.0 equiv., 12.0 mmol, 1.67 mL) and HBTU (1.2 equiv., 4.80 mmol, 1.82 g) were added sequentially, with 2 min between each addition, and the mixture was stirred at rt overnight. The solvent was removed under reduced pressure to afford a residue that was partitioned between EtOAc (50 mL) and KHSO_4_ (1 M, 50 mL). After separation of the phases, the organic phase was thoroughly washed with KHSO_4_ (1 M, 2 × 50 mL), NaHCO_3_ (1 M, 3 × 50 mL), and brine (3 × 50 mL) and then dried with anhydrous MgSO_4_. Filtration followed by removal of the solvent under reduced pressure afforded compound **6** as a diasteroisomeric mixture with a 99% yield. ^1^H NMR (CDCl_3_, 400 MHz) δ): 1.39 (9H, s, O_2_C(C*H*_3_)_3_), 2.79–2.94 (1H, m, β-C*H*_2_ Phe), 2.95–3.06 (1H, m, β-C*H*_2_ Phe), 3.87 and 3.71 (3H, s, OC*H*_3_), 3.42 and 3.52 (1H, brs and d, J = 4.4, O*H*), 4.21–4.44 (1H, m, α-C*H* Phe), 4.72–4.78 (1H, m, α-C*H* Phe(β-O*H*), 4.91–5.04 (1H, m, N*H*), 5.18–5.29 (1H, m, β-C*H* Phe(β-O*H*), 6.80–6.89 (1H, m, N*H*), 7.18.7.35 (10H, m, Ph*H*) ppm.

#### 4.1.16. Synthesis of Boc-L-Phe-Z-ΔPhe-OMe (**7**)

Compound **6** (1.0 equiv., 1.75 g, 3.97 mmol) was dissolved in dry MeCN (5 mL). Boc_2_O (1.20 equiv., 1.04 mg, 4.76 mmol) and DMAP (0.12 equiv., 0.06 mg, 0.48 mmol) were added. The mixture was stirred with a calcium chloride tube. The reaction was monitored using ^1^H NMR. After the disappearance of the starting material was observed, TMG (2% *v*/*v*, 0.1 mL) was added. Once all the intermediates had reacted, the solvent was removed, and the residue was dissolved in ethyl acetate (50 mL). The organic phase was washed with KHSO_4_ 1 M, NaHCO_3_ 1 M and brine (3 × 30 mL each). The organic phase was dried with anhydrous magnesium sulphate, and the solvent was removed under reduced pressure. Compound **7** was obtained in 69% yield. (1.16 g, 2.73 mmol). ^1^H NMR (CDCl_3_, 400 MHz, δ): 1.41 (9H, s, O_2_C(C*H*_3_)_3_), 3.08 (1H, dd, J = 14.0 Hz, 7.2 Hz, β-C*H*_A_CH_B_Ph Phe), 3.21 (1H, dd, J = 14.0 Hz, 6.4 Hz, β-CH_A_C*H*_B_Ph Phe), 3.82 (3H, s, CO_2_C*H*_3_), 4.46–4.55 (1H, m, α-C*H* Phe), 4.97 (1H, br s, N*H*), 7.28–7.42 (11, m, Ph*H*, α-C*H*Phe), 7.68 (1H, br s, N*H*) ppm. ^13^C NMR (CDCl_3_, 100.6 MHz, δ): 28.1 (3 x CH_3_, OC(*C*H_3_)_3_), 37.5 (CH_2_, β-CH_2_ Phe), 52.5 (CH_3_, CO_2_*C*H_3_), 80.3 (C, O*C*(CH_3_)_3_), 123.8 (C, α-*C* ΔPhe), 126.8 (CH, Ph), 128.49 (CH, Ph), 128.53 (CH, Ph), 129.3 (CH, Ph), 129.4 (CH, Ph), 129.7 (C, Ph), 132.7 (CH, β-*C*H ΔPhe), 133.3 (C, Ph), 136.4 (C, Ph), 165.3 (C, *C*=O ΔPhe), 170.3 (C, *C*=O Boc), 171.1 (C, *C*=O Phe) ppm.

#### 4.1.17. Synthesis of Compound **8a**

Compound **7** (2.2 equiv., 1.52 mmol, 644 mg) was dissolved in TFA (2 mL) during 1 h. Ethyl ether was added, and the solvent was evaporated under reduced pressure. Then, we added DMF (5 mL), 2,2′-(1,4-Phenylene)diacetic acid (1.0 equiv., 0.689 mmol, 134 mg), HBTU (2.4 equiv., 0.164 mmol, 622 mg) and Et_3_N (6.0 equiv., 4.13 mmol, 0.576 mL). The reaction was left stirring at room temperature overnight. Water (25 mL) was added to the reaction to precipitate. The solid was filtrated to afford compound **8a** (348 mg, 0.430 mmol, 62%), as a white solid. ^1^H-NMR (400 MHz, DMSO-d_6_, δ): 2.79–2.87 (2H, m, β-C*H*_2_ Phe), 3.10–3.14 (2H, dd, *J* = 4.8 and 14 Hz, β-C*H*_2_ Phe), 3.32–3.41 (4H, m, C*H*_2_ central ring), 3.68 (6H, s, OC*H*_3_), 4.68–4.73 (2H, m, α-C*H* Phe), 6.93 (4H, s, Ar*H* central ring), 7.17–7.37 (18H, m, β-C*H* ΔPhe, Ar*H* Phe, Ar*H* ΔPhe), 7.59–7.61 (4H, m, Ar*H* ΔPhe), 8.23 (2H, d, *J* = 8.0 Hz, N*H* Phe), 9.89 (2H, s, N*H* ΔPhe) ppm. ^13^C-NMR (100.6 MHz, DMSO-d_6_ δ): 37.7 (CH_2_, β-*C*H_2_ Phe), 41.6 (CH_2_, *C*H_2_ central ring), 52.2 (CH_3_, O*C*H_3_), 54.0 (CH, α-*C*H Phe), 125.9 (C), 126.3 (CH, Ar*C*H), 128.1 (CH, Ar*C*H), 128.6 (CH, Ar*C*H), 128.6 (CH, Ar*C*H central ring), 129.2 (CH, Ar*C*H), 129.5 (CH, Ar*C*H), 130.0 (CH, Ar*C*H ΔPhe), 131.9 (CH, β-*C*H ΔPhe), 133.1 (C), 134.1 (C), 137.7 (C), 165.4 (C, *C*=O), 170.2 (C, *C*=O), 171.5 (C, *C*=O) ppm.

#### 4.1.18. Synthesis of Compound **8b**

Compound **7** (2.2 equiv., 1.40 mmol, 600 mg) was dissolved in TFA (2 mL) during 1 h. Ethyl ether was added, and the solvent was evaporated under reduced pressure. Then, we added DMF (5 mL), succinic acid (1.0 equiv., 0.642 mmol, 76 mg), HBTU (2.4 equiv., 1.50 mmol, 584 mg) and Et_3_N (6.0 equiv., 3.85 mmol, 0.537 mL). The reaction was left stirring at room temperature overnight. Water (25 mL) was added to the reaction to precipitate. The solid was filtrated to afford compound **8b** (424 mg, 0.580 mmol, 90%), as a white solid. ^1^H-NMR (400 MHz, DMSO-d_6_, δ): 2.17–2.32 (4H, m, C*H*_2_ succinic), 2.75–2.81 (2H, m, β-C*H*_2_ Phe), 3.05–3.12 (2H, m, β-C*H*_2_ Phe), 3.69 (6H, s, OC*H*_3_), 4.60–4.65 (2H, m, α-C*H* Phe), 7.18–7.38 (18H, m, β-C*H* ΔPhe, Ar*H* ΔPhe, Ar*H* Phe), 7.60–7.64 (4H, m, Ar*H* ΔPhe), 8.23 (2H, d, *J* = 8.4 Hz, N*H* Phe), 9.81 (2H, s, N*H* ΔPhe) ppm. ^13^C-NMR (100.6 MHz, DMSO-d_6_ δ): 25.01 (CH_2_, *C*H_2_ succinic), 36.9 (CH_2_, β-*C*H_2_ Phe), 52.1 (CH_3_, O*C*H_3_), 54.1 (CH, α-*C*H Phe), 125.9 (C), 126.3 (CH, Ar*C*H), 128.0 (CH, Ar*C*H), 128.5 (CH, Ar*C*H), 129.1 (CH, Ar*C*H), 129.4 (CH, Ar*C*H), 130.1 (CH, Ar*C*H), 131.8 (CH, β-*C*H ΔPhe), 133.2 (C), 137.9 (C), 165.3 (C, *C*=O ΔPhe), 171.4 (C, *C*=O), 171.5 (C, *C*=O) ppm.

#### 4.1.19. Synthesis of Compound **8c**

Compound **7** (2.4 equiv., 1.39 mmol, 610 mg) was dissolved in TFA (2 mL) during 1 h. Ethyl ether was added, and the solvent was evaporated under reduced pressure. Then, we added DMF (5 mL), suberic acid (1.0 equiv., 0.580 mmol, 100 mg), HBTU (2.4 equiv., 1.39 mmol, 530 mg) and Et_3_N (6.0 equiv., 4.13 mmol, 0.580 mL). The reaction was left stirring at room temperature overnight. Water (25 mL) was added to the reaction to precipitate. The solid was filtrated the compound to afford compound **8c** (450 mg, 0.570 mmol, 98%), as a beige solid. ^1^H-NMR (400 MHz, DMSO-d_6_, δ): 0.94–1.00 (4H, m, C*H*_2_ suberic), 1.29–1.32 (4H, m, C*H*_2_ suberic), 2.00 (4H, t, *J* = 7.6 Hz, C*H*_2_ suberic), 2.75–2.78 (2H, m, β-C*H*_2_ Phe), 3.02–3.07 (2H, m, β-C*H*_2_ Phe), 3.70 (6H, s, OC*H*_3_), 4.66–4.72 (4H, m, α-C*H* Phe), 7.17–7.36 (18H, m, β-C*H* ΔPhe, Ar*H* ΔPhe, Ar*H* Phe), 7.61–7.64 (4H, m, Ar*H* ΔPhe), 8.01 (2H, d, *J* = 8.4 Hz, N*H* Phe), 9.82 (2H, s, N*H* ΔPhe) ppm. ^13^C-NMR (100.6 MHz, DMSO-d_6_ δ): 25.01 (CH_2_, *C*H_2_ suberic), 28.3 (CH_2_, *C*H_2_ suberic), 35.1 (CH_2_, *C*H_2_ suberic), 37.0 (CH_2_, β-*C*H_2_ Phe), 52.2 (CH_3_, O*C*H_3_), 53.9 (CH, α-*C*H Phe), 125.9 (C), 126.3 (CH, Ar*C*H), 128.0 (CH, Ar*C*H), 128.5 (CH, Ar*C*H), 129.1 (CH, Ar*C*H), 129.4 (CH, Ar*C*H), 130.1 (CH, Ar*C*H), 132.0 (CH, β-*C*H ΔPhe), 133.2 (C), 137.4 (C), 165.4 (C, *C*=O ΔPhe), 170.3 (C, *C*=O Phe), 172.1 (C, *C*=O Suberic) ppm.

#### 4.1.20. Synthesis of Compound **8d**

Compound **7** (2.2 equiv., 1.00 mmol, 420 mg) was dissolved in TFA (2 mL) during 1 h. Ethyl ether was added, and the solvent was evaporated under reduced pressure. Then, we added DMF (5 mL), azelaic acid (0.5 equiv., 0.500 mmol, 90 mg), HBTU (2.0 equiv., 1.00 mmol, 0.380 mg) and Et_3_N (6.0 equiv., 3.00 mmol, 0.620 mL). The reaction was left stirring at room temperature overnight. Water (25 mL) was added to the reaction to precipitate. The solid was filtrated the compound to afford compound **8d** (300 mg, 0.380 mmol, 75%), as a white solid. ^1^H-NMR (400 MHz, DMSO-d_6_, δ): 1.00–1.08 (6H, m, C*H*_2_ azelaic), 1.30–1.45 (4H, m, C*H*_2_ azelaic), 2.00–2.05 (4H, m, C*H*_2_ azelaic), 2.75–2.81 (4H, m, β-C*H*_2_ Phe), 3.07–3.12 (4H, m, β-C*H*_2_ Phe), 3.68 (6H, s, OCH_3_), 4.66–4.72 (2H, α-C*H*_2_ Phe), 7.17–7.36 (18H, m Ar*H* ΔPhe, Ar*H* Phe, β-C*H* ΔPhe), 7.62–7.64 (4H, m, Ar*H* ΔPhe), 8.10 (2H, d, *J* = 8.4 Hz, N*H* Phe), 9.82 (2H, s, N*H* ΔPhe) ppm. ^13^C-NMR (100.6 MHz, DMSO-d_6_ δ): 25.2 (CH_2_, *C*H_2_ azelaic), 28.3 (CH_2_, *C*H_2_ azelaic), 35.2 (CH_2_, *C*H_2_ azelaic), 36.9 (CH_2_, β-*C*H_2_ Phe), 52.2 (CH_3_, O*C*H_3_), 53.8 (CH, α-*C*H Phe), 125.9 (C), 126.3 (CH, Ar*C*H), 128.0 (CH, Ar*C*H), 128.5 (CH, Ar*C*H), 129.2 (CH, Ar*C*H), 129.4 (CH, Ar*C*H), 130.1 (CH, Ar*C*H ΔPhe), 131.9 (CH, β-*C*H ΔPhe), 133.2 (C), 137.9 (C), 165.4 (C, *C*=O), 171.7 (C, *C*=O), 172.3 (C, *C*=O) ppm.

#### 4.1.21. Synthesis of Compound **2a**

Compound **8a** (0.430 mmol, 348 mg) was dissolved in 1,4-dioxane (10 mL) and NaOH (1 M) (6.00 equiv., 2.50 mL). The reaction was monitored by TLC. When all the starting material was consumed, the organic solvent was removed under reduced pressure, and the reaction mixture was acidified to pH 3 with KHSO_4_ (1 M). The solid precipitate was filtered to afford compound **2a** (319 mg, 0.409 mmol, 95%), as a white solid. ^1^H-NMR (400 MHz, DMSO-d_6_, δ): 2.76–2.83 (2H, m, β-C*H*_2_ Phe), 3.12–3.15 (2H, m, β-C*H*_2_ Phe), 3.32–3.41 (4H, q, *J* = 13.2 and 14 Hz, C*H*_2_ central ring), 4.67–4.73 (2H, m, α-C*H* Phe), 6.89 (4H, s, Ar*H* central ring), 7.16–7.32 (18H, m, β-C*H* ΔPhe, Ar*H* Phe, Ar*H* ΔPhe), 7.58–7.60 (4H, m, Ar*H* ΔPhe), 8.36 (2H, d, *J* = 8.0 Hz, N*H* Phe), 9.71 (2H, s, N*H* ΔPhe), 12.7 (2H, s, CO_2_*H*) ppm. ^13^C-NMR (100.6 MHz, DMSO-d_6_ δ): 37.2 (CH_2_, β-*C*H_2_ Phe), 41.6 (CH_2_, *C*H_2_ central ring), 54.1 (CH, α-*C*H Phe), 126.3 (CH, Ar*C*H), 126.6 (C), 128.0 (CH, Ar*C*H), 128.5 (CH, Ar*C*H), 128.6 (CH, Ar*C*H central ring), 129.0 (CH, Ar*C*H), 129.2 (CH, Ar*C*H), 130.0 (CH, Ar*C*H ΔPhe), 131.7 (CH, β-*C*H ΔPhe), 133.6 (C), 134.0 (C), 137.8 (C), 166.2 (C, *C*=O), 170.4 (C, *C*=O), 171.1 (C, *C*=O) ppm. HRMS (ESI): *m*/*z*: [M+H]^+^ calcd. For C_46_H_42_N_4_O_8_ 779.3003; found 779.3077. HRMS (ESI): [M+H] calcd. for C_46_H_43_N_4_O_8_ 779.3081; found 779.3064.

#### 4.1.22. Synthesis of Compound **2b**

Compound **8b** (0.580 mmol, 424 mg) was dissolved in 1,4-dioxane (10 mL) and NaOH (1 M) (6.00 equiv., 3.48 mmol, 3.48 mL). The reaction was monitored by TLC. When all the starting material was consumed, the organic solvent was removed under reduced pressure, and the reaction mixture was acidified to pH 3 with KHSO_4_ (1 M). The solid precipitate was filtered to afford bolaamphiphile **2b** (267 mg, 0.380 mmol, 65%), as a yellow solid. ^1^H-NMR (400 MHz, DMSO-d_6_, δ): 2.13–2.34 (4H, m, C*H*_2_ succinic), 2.73–2.79 (2H, m, β-C*H*_2_ Phe), 3.08–3.13 (2H, m, β-C*H*_2_ Phe), 4.57–4.63 (2H, m, α-C*H* Phe), 7.16–7.35 (18H, m, β-C*H* ΔPhe, Ar*H* ΔPhe, Ar*H* Phe), 7.54–7.56 (4H, m, Ar*H* ΔPhe), 8.23 (2H, d, *J* = 8.4 Hz, N*H* Phe), 9.60 (2H, s, N*H* ΔPhe) ppm. ^13^C-NMR (100.6 MHz, DMSO-d_6_ δ): 30.70 (CH_2_, *C*H_2_ succinic), 36.9 (CH_2_, β-*C*H_2_ Phe), 54.3 (CH, α-*C*H Phe), 126.1 (CH, Ar*C*H), 127.4 (C), 128.0 (CH, Ar*C*H), 128.3 (CH, Ar*C*H), 128.8 (CH, Ar*C*H), 129.2 (CH, Ar*C*H), 129.8 (CH, Ar*C*H ΔPhe), 130.3 (CH, β-*C*H ΔPhe), 134.0 (C), 130.1 (C), 166.5 (C, *C*=O ΔPhe), 170.8 (C, *C*=O), 171.6 (C, *C*=O) ppm. HRMS (ESI): [M+H] calcd. for C_40_H_39_N_4_O_8_ 703.2768; found 703.2783.

#### 4.1.23. Synthesis of Compound **2c**

Compound **8c** (0.520 mmol, 410 mg) was dissolved in 1,4-dioxane (5.0 mL) and NaOH (1 M) (6.00 equiv., 3.12 mmol, 3.12 mL). The reaction was monitored by TLC. When all the starting material was consumed, the organic solvent was removed under reduced pressure, and the reaction mixture was acidified to pH 3 with KHSO_4_ (1 M). The solid precipitate was filtered to afford bolaamphiphile **2c** (320 mg, 0.420 mmol, 81%), as a white solid. ^1^H-NMR (400 MHz, DMSO-d_6_, δ): 0.99 (4H, d, *J* = 4.0 Hz, C*H*_2_ suberic), 1.29 (4H, t, *J* = 6.8 Hz, C*H*_2_ suberic), 1.97 (4H, t, *J* = 7.2 Hz, C*H*_2_ suberic), 2.72–2.78 (2H, m, β-C*H*_2_ Phe), 3.08–3.12 (2H, dd, *J* = 3.2 and 13.2 Hz, β-C*H*_2_ Phe), 4.63–4.70 (4H, m, α-C*H* Phe), 7.22–7.33 (18H, m, β-C*H* ΔPhe, Ar*H* ΔPhe, Ar*H* Phe), 7.57–7.59 (4H, m, Ar*H* ΔPhe), 8.16 (2H, d, *J* = 8.4 Hz, N*H* Phe), 9.65 (2H, s, N*H* ΔPhe) ppm. ^13^C-NMR (100.6 MHz, DMSO-d_6_ δ): 25.05 (CH_2_, *C*H_2_ suberic), 28.2 (CH_2_, *C*H_2_ suberic), 35.1 (CH_2_, *C*H_2_ suberic), 37.0 (CH_2_, β-*C*H_2_ Phe), 54.1 (CH, α-*C*H Phe), 126.2 (CH, Ar*C*H), 127.2 (C), 128.0 (CH, Ar*C*H), 128.3 (CH, Ar*C*H), 128.9 (CH, Ar*C*H), 129.2 (CH, Ar*C*H), 130.0 (CH, Ar*C*H), 130.8 (CH, β-*C*H ΔPhe), 134.0 (C), 138.1 (C), 166.4 (C, *C*=O), 171.1 (C, *C*=O), 172.3 (C, *C*=O) ppm. HRMS (ESI): [M+H] calcd. for C_44_H_47_N_4_O_8_ 759.3394; found 759.3408.

#### 4.1.24. Synthesis of Compound **2d**

Compound **8d** (0.380 mmol, 300 mg) was dissolved in 1,4-dioxane (5 mL) and NaOH (1 M) (6.00 equiv., 2.20 mL). The reaction was monitored by TLC. When all the starting material was consumed, the organic solvent was removed under reduced pressure, and the reaction mixture was acidified to pH 3 with KHSO_4_ (1 M). The solid precipitate was filtered to afford compound **2d** (240 mg, 0.310 mmol, 83%), as a white solid. ^1^H-NMR (400 MHz, DMSO-d_6_, δ): 0.98–1.06 (6H, m, C*H*_2_ azelaic), 1.28–1.35 (4H, m, C*H*_2_ azelaic), 2.00 (4H, t, *J* = 7.2 Hz C*H*_2_ azelaic), 2.73–2.80 (2H, m, β-C*H*_2_ Phe), 3.08–3.13 (2H, dd, *J* = 3.6 and 13.6 Hz, β-C*H*_2_ Phe), 4.64–4.70 (4H, m, α-C*H* Phe), 7.15–7.36 (18H, m, β-C*H* ΔPhe, Ar*H* ΔPhe, Ar*H* Phe), 7.58–7.61 (4H, m, Ar*H* ΔPhe), 8.40 (2H, d, *J* = 8.4 Hz, N*H* Phe), 9.65 (2H, s, N*H* ΔPhe) ppm. ^13^C-NMR (100.6 MHz, DMSO-d_6_ δ): 25.1 (CH_2_, *C*H_2_ azelaic), 28.5 (CH_2_, *C*H_2_ azelaic), 35.2 (CH_2_, *C*H_2_ azelaic), 37.0 (CH_2_, β-*C*H_2_ Phe), 54.0 (CH, α-*C*H Phe), 126.2 (CH, Ar*C*H), 126.8 (C), 128.0 (CH, Ar*C*H), 128.4 (CH, Ar*C*H), 129.0 (CH, Ar*C*H), 129.1 (CH, Ar*C*H), 130.0 (CH, Ar*C*H), 131.1 (CH, β-*C*H ΔPhe), 133.7 (C), 138.0 (C), 166.3 (C, *C*=O), 171.2 (C, *C*=O), 172.3 (C, *C*=O) ppm. HRMS (ESI): [M+H] calcd. for C_45_H_49_N_4_O_8_ 773.3550; found 773.3573.

### 4.2. Critical Aggregation Concentration (CAC)

The determination of the critical aggregation concentration value was achieved for all the bolaamphiphiles by fluorescence titration of the dye 8-anilino-1-napthalene sulfonic acid ammonium salt (ANS) with increasing amounts of peptide solution. The fluorescence spectra were collected at room temperature on aShimadzu, RF5301PC (Kyoto, Japan). All the spectra, acquired in a quartz cell with a 1 cm path length at room temperature, were recording using the following settings: excitation and emission bandwidths = 5 nm, excitation wavelength = 350 nm. The maximum fluorescence intensity was plotted against peptide concentration and CAC was found in the intersection of the two linear regressions.

### 4.3. Computational Methods

#### 4.3.1. Initial Setup

Molecular structures of compounds **3**, **1a**, and **1e** were built in UCSF Chimera [42] and optimized in Gaussian09 [43] at the B3LYP/6-31++G(d,p) [44] level with Polarizable Continuum Model solvent representation [45]. Atomic partial charges were assigned using the Restrained Electrostatic Potential (RESP) method [46], based on HF/6-31++G(d,p) single-point calculations performed on the optimized geometries (mol2 files available upon request). Initial configurations were generated using PACKMOL v18.169 [47], placing 10 molecules in a 75 Å cubic box with an 8 Å distance tolerance. Systems were solvated with ~10,500 OPC water molecules [48] using the LEaP module in AMBER18 (University pf California, San Francisco, CA, USA) [49], with no counterions required to neutralize the systems.

#### 4.3.2. Molecular Dynamics

Molecular dynamics (MD) simulations were conducted using the GPU-enabled PMEMD engine in AMBER18 [49] with the GAFF2 force field [50]. Following two stages of energy minimization, the systems were equilibrated for 500 ps in the NVT ensemble using Langevin dynamics, with small restraints of 10 kcal mol^−1^ to heat the system. Subsequently, 300 ns production runs were performed at 310 K in the NPT ensemble with a Langevin collision frequency of 1 ps^−1^. Pressure was maintained at 1 atm using isotropic position scaling with a 2 ps relaxation time under periodic boundary conditions. A 2 fs integration time step was employed, with hydrogen-containing bonds constrained via the SHAKE algorithm [51]. Electrostatic interactions were evaluated using the Particle Mesh Ewald (PME) method [52] with a 10 Å cutoff. The MD trajectories were centered back to the primary box resorting to the CPPTRAJ [53] module implemented in AMBER18.

### 4.4. Preparation of Hydrogels

For the formation of the hydrogel, the compounds were weighed into a sample vial (**1a**, 4 mg; **1c**, 5 mg; **1e**, 4 mg; **2a**, 4 mg; **2a**, 4 mg; **2c**, 4 mg and **2d**, 4 mg) and water (1 mL) was added. While stirring, NaOH (1 M) was added till pH 10. The mixture was sonicated and then GdL was added (**1a**, **1c** and **1e** used 6 mg of GdL; **2b** used 4 mg of GdL; **2c** used 8 mg of GdL; and **2d** used 1.2 mg of GdL). The solutions were left standing overnight to form the hydrogel.

### 4.5. Transmission Electron Microscope (TEM)

Carbon-coated 400-mesh copper grids were glow-discharged prior to use. A 5 µL aliquot of each sample was deposited onto the grids, and excess solution was removed after 60 s. The grids were stained with 2% (*v*/*v*) uranyl acetate and air-dried. TEM images were recorded using a Morgagni 268 ((Hillsboro, OR, USA) microscope operating at a higher voltage (HV) of 80 kV, with a filament setting of 2.

### 4.6. Rheology

Viscoelastic characterization was carried out using a stress-controlled Anton Paar MCR300 (Anton Paar GmbH, Graz, Austria) rotational rheometer. Samples were loaded into a Couette cell (1 mL, 0.5 mm gap) at 25 °C and pre-sheared at 5 s^−1^ for 1 min. Gelation kinetics were monitored for 10 h under oscillatory shear (1 Hz, 0.001% or 0.0001% strain), recording G′ and G″ every second. Frequency sweeps (100–0.01 Hz) and strain sweeps (0.001–100%) were subsequently performed and both storage (G′) and loss (G″) shear moduli were measured.

### 4.7. Determination of Gel–Sol Transition Temperature

The gel–sol transition temperature of hydrogel **2a** was determined using the tube inversion method under controlled heating conditions. Hydrogel samples of identical volume were transferred into identical glass tubes and equilibrated at 25 °C prior to analysis. The tubes were then immersed in an oil bath and heated from 25 to 100 °C at a constant rate of 0.5 °C min^−1^. At defined temperature intervals, each tube was carefully removed and inverted to assess the structural integrity of the gel. Samples were considered to remain in the gel state if no visible flow was observed within 30 s after inversion. The gel–sol transition temperature was defined as the lowest temperature at which the sample exhibited flow under these conditions.

### 4.8. Biological Studies

HaCaT human keratinocytes (ATCC) were cultured in DMEM supplemented with 10% FBS and 1% penicillin/streptomycin at 37 °C under 5% CO_2_. Cells were seeded in 96-well plates (1.5 × 10^4^ cells per well) and allowed to adhere for 24 h, followed by incubation with the indicated concentrations of the compounds for 24 h. Cell viability was assessed using the MTT assay by measuring the reduction of MTT to formazan after 2 h. Absorbance was recorded at 570 nm using a Multiskan GO microplate reader (Thermo Fisher Scientific, Waltham, MA, USA). Results are expressed as percentage of control and represent mean ± SEM of at least three independent experiments performed in triplicate. Statistical analysis of biological assays was performed using ANOVA to compare controls and experimental groups. Outliers were detected using Grubbs’ test. Results are expressed as mean ± SD of at least three independent experiments. Analyses were conducted in GraphPad Prism 9.0, with *p* < 0.05 considered significant.

### 4.9. Drug Delivery Assays

Hydrogel **2a** was prepared as previously described to obtain 1 mL hydrogels containing the same concentration of hydrogelator and methotrexate (MTX, 300 µM) (manufacturer, city, and country). After equilibration overnight, 1.5 mL of phosphate-buffered saline (PBS, pH 7.4) was carefully layered onto the hydrogel surface. Aliquots (150 µL) of the supernatant were collected at predetermined time points (0.5, 1, 2, 4, 6, 24, 48, and 72 h). After each sampling, the removed volume was immediately replaced with an equal volume of fresh PBS to maintain constant conditions. MTX concentration in each aliquot was determined by measuring its absorbance at λ_max_ = 303 nm using a microplate reader. Experiments were performed in triplicate, and mean drug release (%) was plotted versus time.

## Figures and Tables

**Table 1:** Calculated critical aggregation concentration and calculated LogP values for the dehydropeptide-based bolaamphiphiles.

Compound	CAC (µM)	cLog P ^1^
**1a**	51	3.78
**1b**	117	2.62
**1c**	39	2.80
**1d**	90	1.36
**1e**	153	3.38
**1f**	123	3.88
**2a**	47	5.71
**2b**	54	4.27
**2c**	86	6.29
**2d**	66	6.80

^1^ cLog P obtained from https://molinspiration.com/ (accessed on 15 September 2025).

**Table 2:** Critical gelation concentration (CGC) of compounds **1a**–**f** and **2a**–**d**.

Compound	CGC (wt%/mM)	GdL (wt%)	pH
**1a**	No hydrogel	---	---
**1b**	No hydrogel	---	---
**1c**	0.4/5.14	0.6	5
**1d**	No hydrogel	---	---
**1e**	0.5/6.30	0.6	5
**1f**	No hydrogel	---	---
**2a**	0.4/5.14	0.6	5
**2b**	0.4/5.70	0.4	6
**2c**	0.4/5.27	0.8	5
**2d**	0.4/5.17	1.2	4

**Table 3:** Rheological characterization of the supramolecular hydrogels formed by compounds **1c**, **1e**, and **2a**–**d**, including the storage modulus (G′), loss modulus (G″), and strain at mechanical breakdown.

Hydrogel from	G′ (Pa)	G″ (Pa)	Strain (%)
**1c**	2.23 × 10^4^	9.54 × 10^2^	30.6
**1e**	3.74 × 10^4^	1.26 × 10^3^	1.28
**2a**	2.50 × 10^4^	5.63 × 10^3^	8.90
**2b**	2.74 × 10^4^	2.98 × 10^2^	4.81
**2c**	8.05 × 10^3^	4.67 × 10^2^	144
**2d**	7.07 × 10^4^	4.55 × 10^4^	4.26

**Table 4:** Storage modulus (G′) of the hydrogels before and after mechanical breakdown and corresponding recovery relative to the initial G′ value.

Hydrogel from	Before Breaking G′ (Pa)	After Breaking G′ (Pa)	Recovery (%) ^1^
**1c**	2.23 × 10^4^	1.04 × 10^3^	5
**1e**	3.74 × 10^4^	2.69 × 10^4^	72
**2a**	2.50 × 10^4^	2.90 × 10^4^	110
**2b**	2.74 × 10^4^	2.83 × 10^4^	103
**2c**	8.05 × 10^3^	1.62 × 10^2^	2
**2d**	7.07 × 10^4^	2.65 × 10^4^	37

^1^ Recovery expressed as a percentage of the initial G′.

**Table 5:** Best fit parameters for the release profile of MTX from hydrogel **2a** to the Korsmeyer–Peppas model.

Korsmeyer–Peppas		
*k*	*n*	R^2^
0.0990	0.323	0.990

## Data Availability

The original contributions presented in this study are included in the article/Supplementary Material. Further inquiries can be directed to the corresponding authors.

## Supplementary Materials

The following supporting information can be downloaded at https://www.mdpi.com/article/10.3390/gels12040306/s1. Scheme S1. Synthesis of symmetrical bolaamphiphiles based on dehydrodipeptides **1a**–**f**. (i) 1. Boc_2_O, DMAP, dry MeCN, 2. TMG; (ii) 1. TFA, 2. HBTU, Et_3_N, DMF; (iii) 1. NaOH (1 M), 1,4-dioxane, 2. K_2_SO_4_ (1 M). Scheme S2. Synthesis of symmetrical bolaamphiphiles based on dehydrodipeptides **2a**–**d**. (i) 1. Boc_2_O, DMAP, dry MeCN, 2. TMG; (ii) 1. TFA, 2. HBTU, Et_3_N, DMF; (iii) 1. NaOH (1 M), 1,4-dioxane, 2. K_2_SO_4_ (1 M). Figure S1. Determination of the critical aggregation concentration (CAC) for bolaamphiphiles **1a** (A, B), **1b** (C, D), **1c** (E, F), **1d** (G, H), **1e** (I, J), **1f** (K, L), **2a** (M, N), **2b** (O, P), **2c** (Q, R). A, C, E, G, I, K, M and O—fluorescence intensity spectra (excitation wavelength = 350 nm) for bolaamphiphiles in a range of concentrations. B, D, F, H, J, L, N, P and R—Plot of fluorescence intensity versus concentration. Figure S2. Representative interactions for peptides **3**. π-interactions (sandwich and PD) are represented as dashed lines and hydrogen bonds with dotted lines. Figure S3. Representative interactions for peptides **1a**. π-interactions (sandwich, T-shape, and PD) are represented as dashed lines. Figure S4. Representative interactions for peptides 1e. Hydrogen bonds are represented as dotted lines. Figure S5. Microscope images from peptide 1a, 1b, 1d and 1f at 0.4 wt%. Figure S6. Elastic and viscous modulus during the kinetic process of gelation for hydrogelators under study: (A) **1c** (0.4 wt%), (B) **1e** (0.5 wt%), (C) **2a** (0.4 wt%), (D) **2b** (0.4 wt%), (E) **2c** (0.4 wt%) and (F) **2d** (0.4 wt%). Figure S7. Frequency dependence of the shear elastic (G′) and loss (G″) moduli for the hydrogels in study: (A) **1c** (0.4 wt%), (B) **1e** (0.5 wt%), (C) **2b** (0.4 wt%), (D) **2c** (0.4 wt%) and (E) **2d** (0.4 wt%). Figure S8. Strain dependence of the shear elastic (G′) and loss (G″) modulus for hydrogels under study: (A) **1c** (0.4 wt%), (B) **1e** (0.5 wt%), (C) **2b** (0.4 wt%), (D) **2c** (0.4 wt%) and (E) **2e** (0.4 wt%). Figure S9. Elastic and viscous modulus during the second kinetic process of gelation for hydrogelators under study: (A) **1c**, (B) **1e**, (C) **2b**, (D) **2c** and (E) **2d**. Figure S10. Sol-gel transition temperature of hydrogel **2a**. (A) Hydrogel at room temperature. (B) hydrogel after heating at 95°C. Figure S11. Cumulative release of MTX from hydrogel **2a** fitted to the Peppas-Sahlin Model (A) and Weilbull Model (B). Results presented as mean and standard deviation of three replicate assay. Table S1. Parameters obtained by fitting the release profiles of drug-load hydrogel **2a** to the Peppas-Sahlin Model and Weilbull Model, with the respective coefficients of determination (R^2^). Figure S12. ^1^H NMR spectrum of compound **1a** in DMSO-d_6_. Figure S13. ^13^C NMR spectrum of compound **1a** in DMSO-d_6_. Figure S14. ^1^H NMR spectrum of compound **1b** in DMSO-d_6_. Figure S15. ^13^C NMR spectrum of compound **1b** in DMSO-d_6_. Figure S16. ^1^H NMR spectrum of compound **1c** in DMSO-d_6_. Figure S17. ^13^C NMR spectrum of compound **1c** in DMSO-d_6_. Figure S18. ^1^H NMR spectrum of compound **1d** in DMSO-d_6_. Figure S19. ^13^C NMR spectrum of compound **1d** in DMSO-d_6_. Figure S20. ^1^H NMR spectrum of compound **1e** in DMSO-d_6_. Figure S21. ^13^C NMR spectrum of compound **1e** in DMSO-d_6_. Figure S22. ^1^H NMR spectrum of compound **1f** in DMSO-d_6_. Figure S23. ^13^C NMR spectrum of compound **1f** in DMSO-d_6_. Figure S24. ^1^H NMR spectrum of compound **2a** in DMSO-d_6_. Figure S25. ^13^C NMR spectrum of compound **2a** in DMSO-d_6_. Figure S26. ^1^H NMR spectrum of compound **2b** in DMSO-d_6_. Figure S27. ^13^C NMR spectrum of compound **2b** in DMSO-d_6_. Figure S28. ^1^H NMR spectrum of compound **2c** in DMSO-d_6_. Figure S29. ^13^C NMR spectrum of compound **2c** in DMSO-d_6_. Figure S30. ^1^H NMR spectrum of compound **2d** in DMSO-d_6_. Figure S31. ^13^C NMR spectrum of compound **2d** in DMSO-d_6_.
