# Supplementary material for: Structure–Property Relationships in Symmetrical Bolaamphiphilic Dehydrodipeptides: Self-Assembled Injectable Hydrogels for Anticancer Drug Delivery
Source: Gels. 2026 Apr 3;12(4):306. doi: 10.3390/gels12040306 (PMC13115458; doi:10.3390/gels12040306)
Supplement: Supplementary file 1 [file gels-12-00306-s001.zip › gels-4199590-supplementary.pdf]

Structure–Property Relationships in Symmetrical Bolaamphiphilic Dehydrodipeptides: Self-Assembled Injectable Hydrogels for Anticancer Drug Delivery

Carolina Amorim [1], André Carvalho [1], Pedro R. Figueiredo [2], Alexandra T.P Carvalho [2,3], Loic Hilliou [4], David M. Pereira [5], Helena Azevedo [6], José A. Martins [1,*] and Paula M.T Ferreira [1,*]

[1] Chemistry Centre, University of Minho (CQ-UM),4710-057 Braga, Portugal; carolinaamorim753@gmail.com (C.A.); id9569@alunos.uminho.pt (A.C.); jmartins@quimica.uminho.pt (J.A.M.); pmf@quimica.uminho.pt (P.M.T.F.)
[2] CNC—Center for Neuroscience and Cell Biology, Institute for Interdisciplinary Research (IIIUC), University of Coimbra, 3004-504 Coimbra, Portugal; pmrfigueiredo@cnc.uc.pt (P.R.F.)
[3] Almac Sciences, Department of Biocatalysis and Isotope Chemistry, Almac House, 20 Seagoe Industrial Estate, Craigavon BT63 5QD, UK; apirescarvalho@gmail.com (A.T.P.C.)
[4] Institute for Polymers and Composites, University of Minho, 4800-058 Guimarães, Portugal; loic@dep.uminho.pt (L.H)
[5] REQUIMTE/LAQV, Laboratório de Farmacognosia, Departamento de Química, Faculdade de Farmácia, Universidade do Porto, R. Jorge Viterbo Ferreira, n 228, 4050-313 Porto, Portugal; dpereira@ff.up.pt (D.M.P.)
[6] i3S - Instituto de Investigação e Inovação em Saúde, Universidade do Porto, Portugal; INEB - Instituto de Engenharia Biomédica, Universidade do Porto, Rua Alfredo Allen, 208, 4200-180 Porto, Portugal
* pmf@quimica.uminho.pt (PMTF); jmartins@quimica.uminho.pt (JAM).

1. Synthesis of bolaamphiphiles

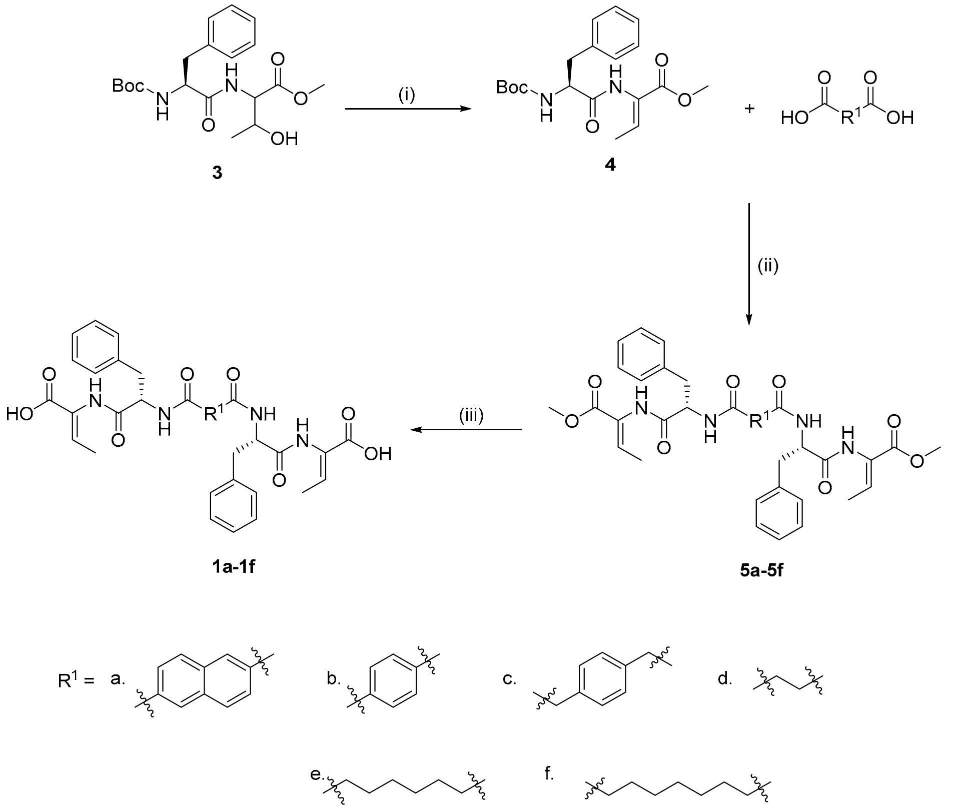

Scheme S1. Synthesis of symmetrical bolaamphiphiles based on dehydrodipeptides **1a-f**. (i) 1. Boc_2O, DMAP, dry MeCN, 2. TMG; (ii) 1. TFA, 2. HBTU, Et_3N, DMF; (iii) 1. NaOH (1M), 1,4-dioxane, 2. K_2SO_4 (1M).

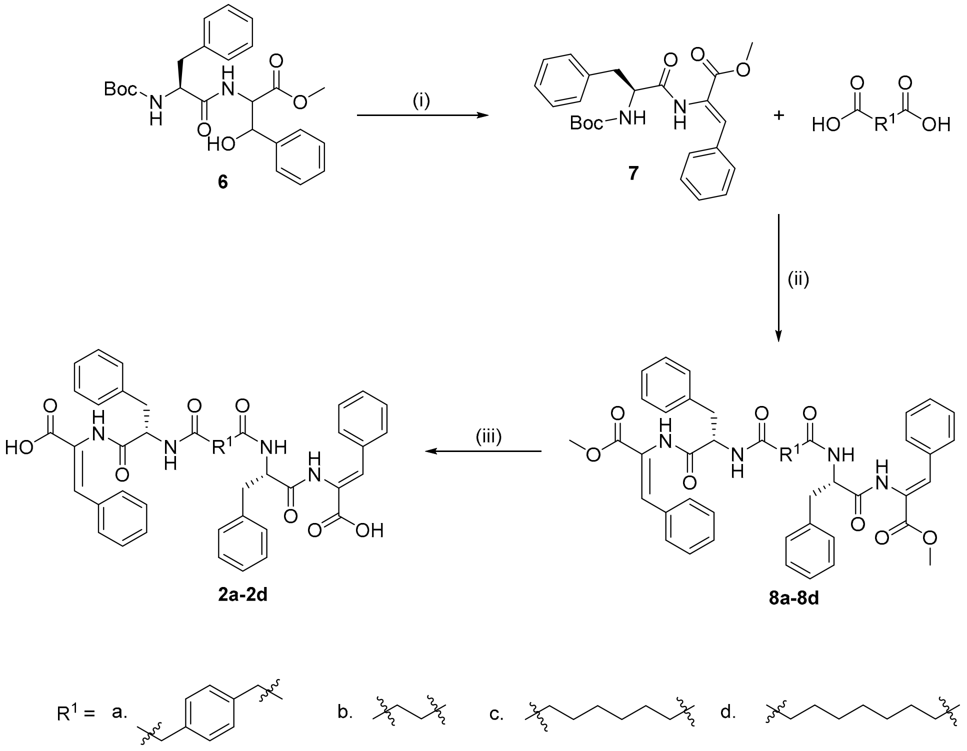

Scheme S2. Synthesis of symmetrical bolaamphiphiles based on dehydrodipeptides **2a-d**. (i) 1. Boc_2O, DMAP, dry MeCN, 2. TMG; (ii) 1. TFA, 2. HBTU, Et_3N, DMF; (iii) 1. NaOH (1M), 1,4-dioxane, 2. K_2SO_4 (1M).

2. Critical aggregation concentration

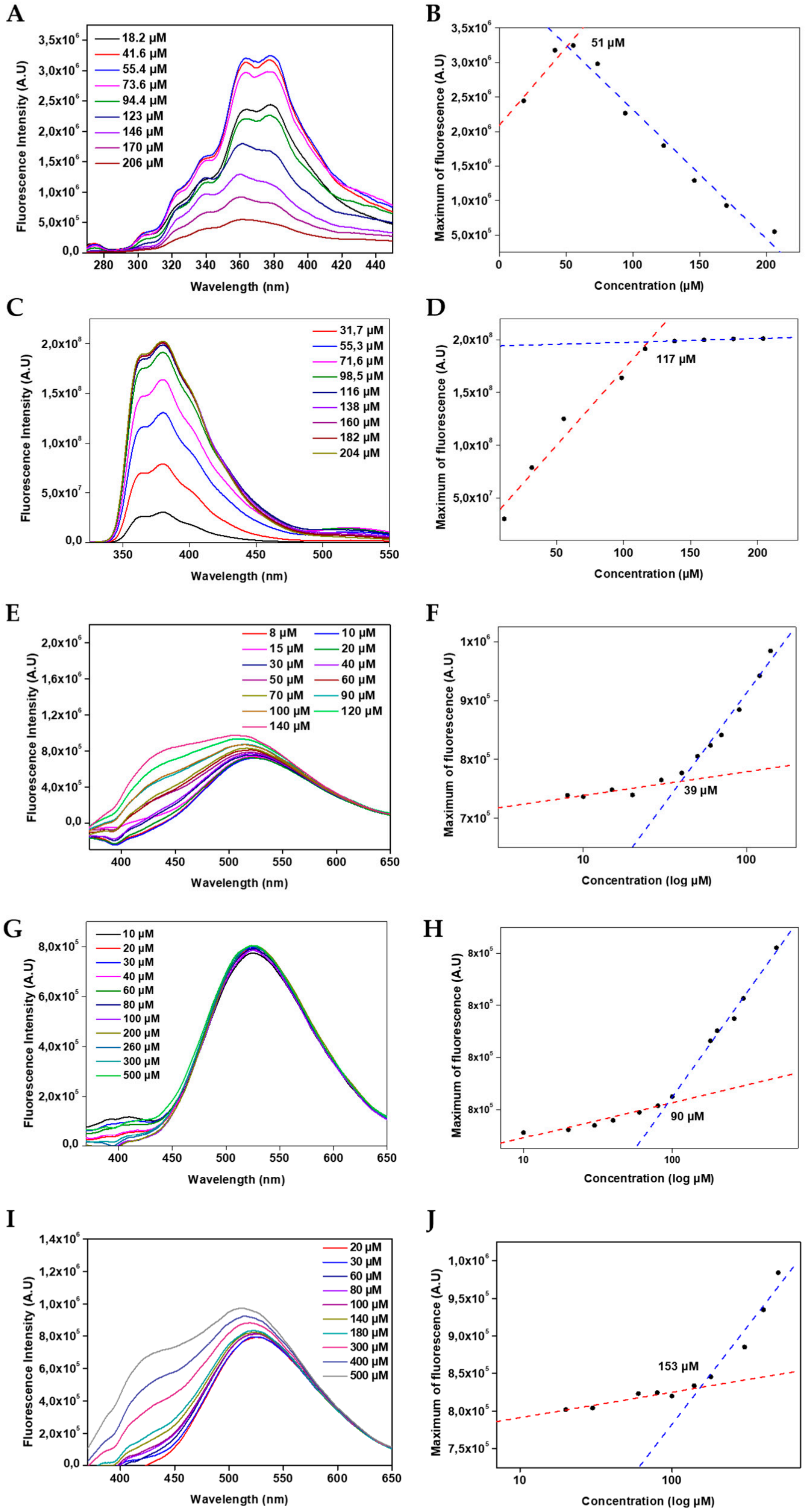

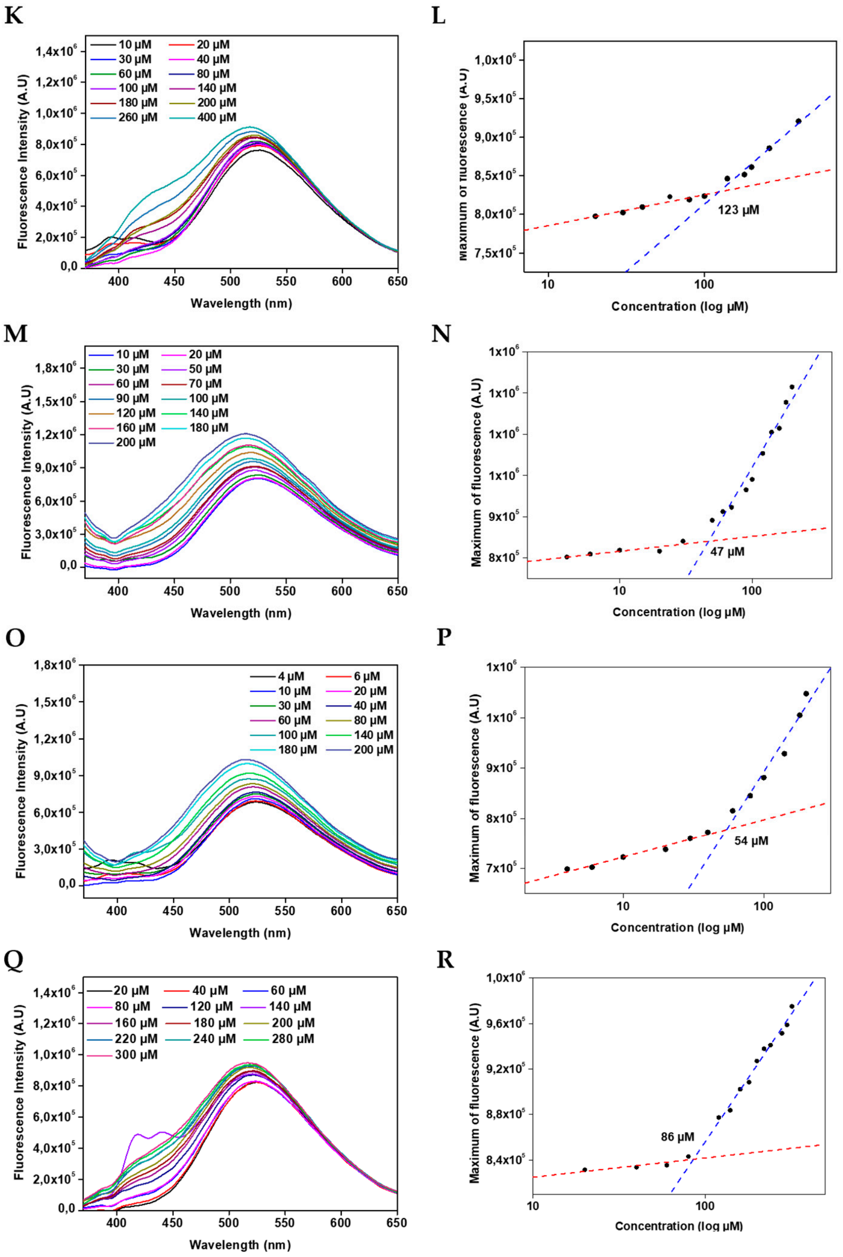

Figure S1. Determination of the critical aggregation concentration (CAC) for bolaamphiphiles **1a** (A, B), **1b** (C, D), **1c** (E, F), **1d** (G, H), **1e** (I, J), **1f** (K, L), **2a** (M, N), **2b** (O, P), **2c** (Q, R). A, C, E, G, I, K, M and O - fluorescence intensity spectra (excitation wavelength = 350 nm) for bolaamphiphiles in a range of concentrations. B, D, F, H, J, L, N, P and R - Plot of fluorescence intensity versus concentration.

3. **Representative intra- and intermolecular interactions**

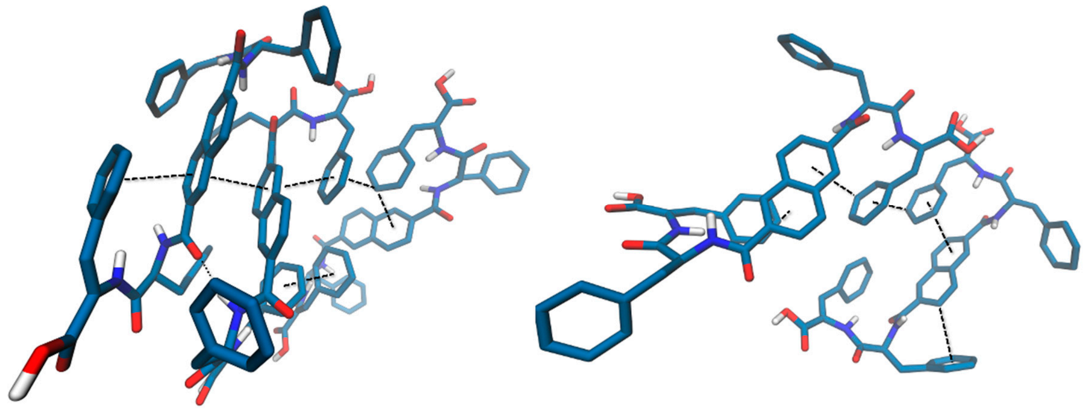

Figure S2. Representative interactions for peptides **3**. π-interactions (sandwich and PD) are represented as dashed lines and hydrogen bonds with dotted lines.

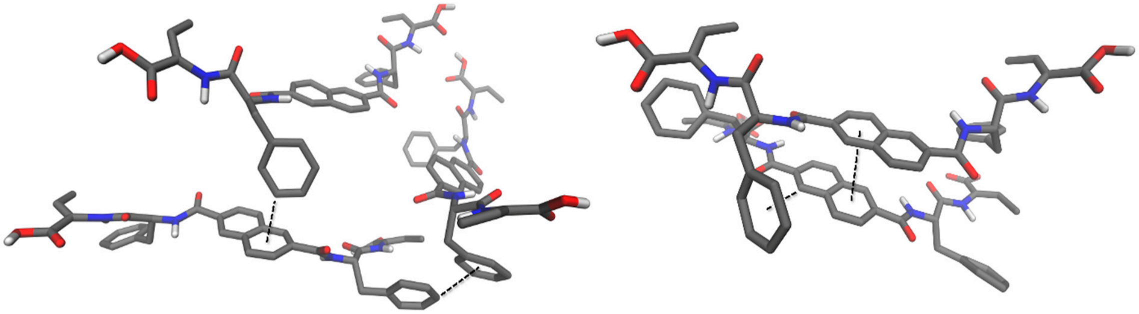

Figure S3. Representative interactions for peptides **1a**. π-interactions (sandwich, T-shape, and PD) are represented as dashed lines.

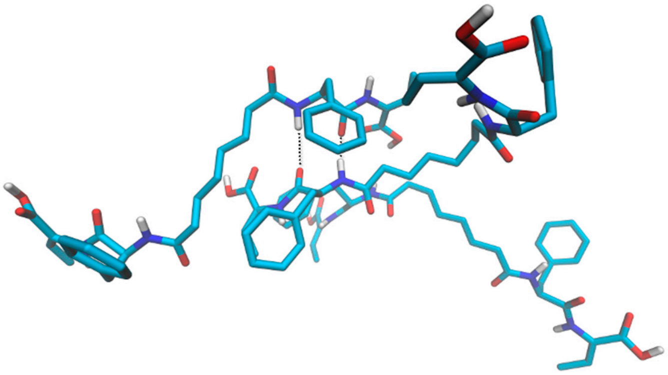

Figure S4. Representative interactions for peptides **1e**. Hydrogen bonds are represented as dotted lines.

4. **Evaluation of the nanostructures from the peptide that don't give hydrogel**

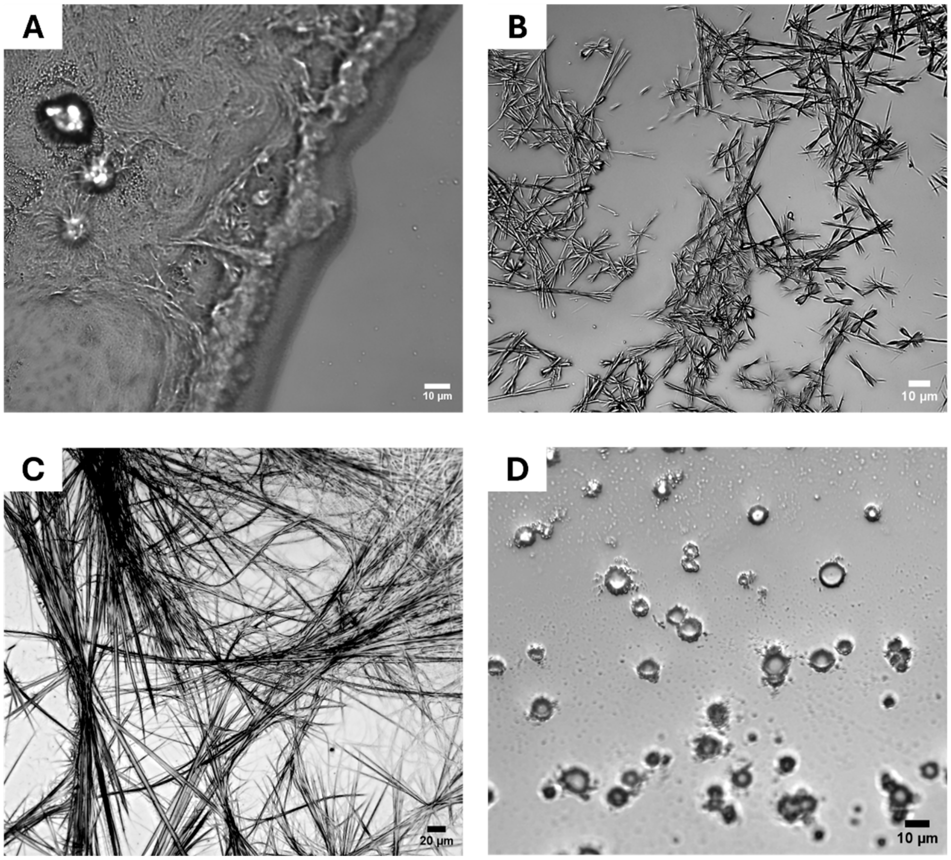

Figure S5. Microscope images from peptide **1a**, **1b**, **1d** and **1f** at 0.4wt%.

5. Rheology

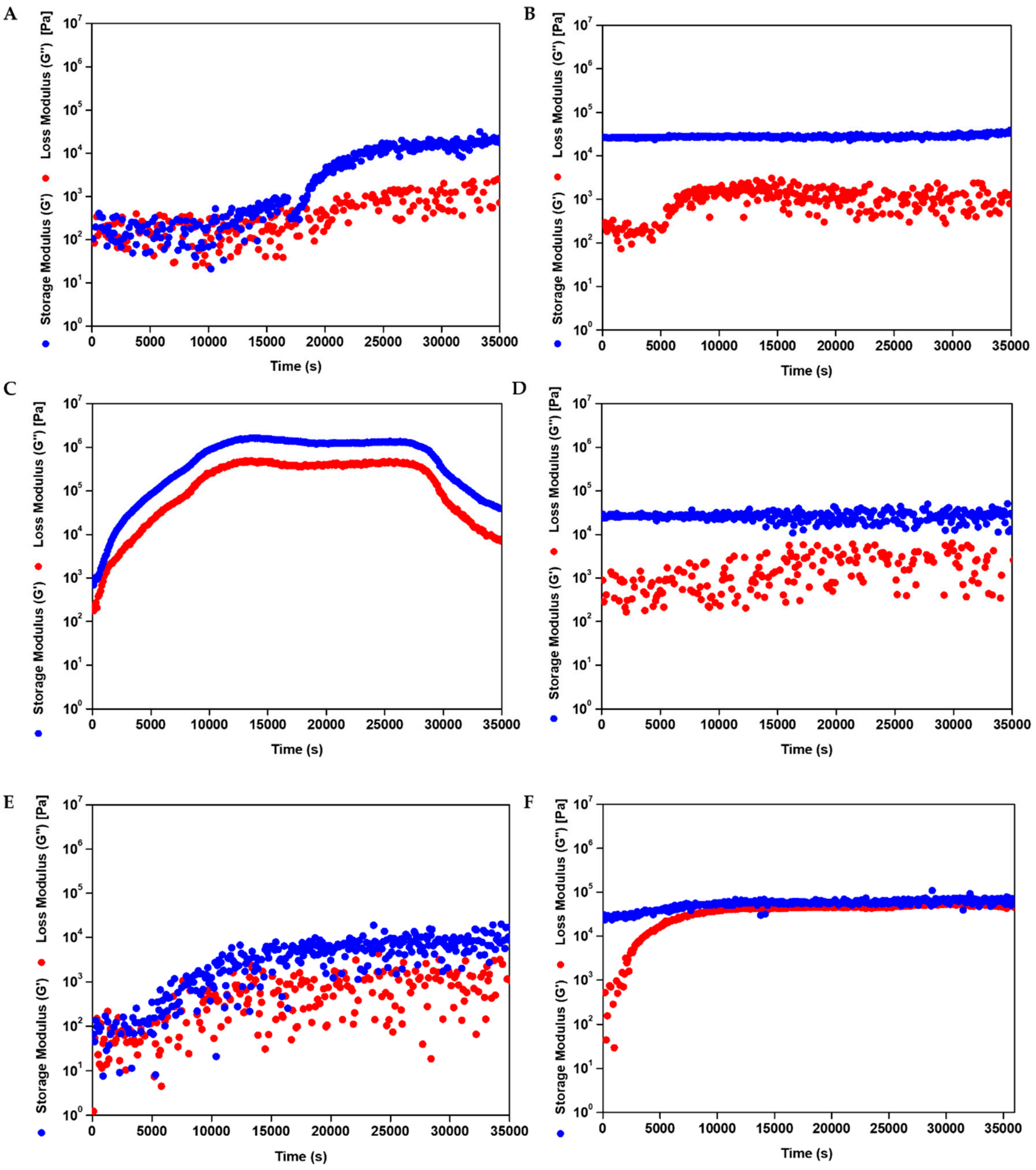

Figure S6. Elastic and viscous modulus during the kinetic process of gelation for hydrogelators under study: (A) **1c** (0.4 wt%), (B) **1e** (0.5 wt%), (C) **2a** (0.4 wt%), (D) **2b** (0.4 wt%), (E) **2c** (0.4 wt%) and (F) **2d** (0.4 wt%).

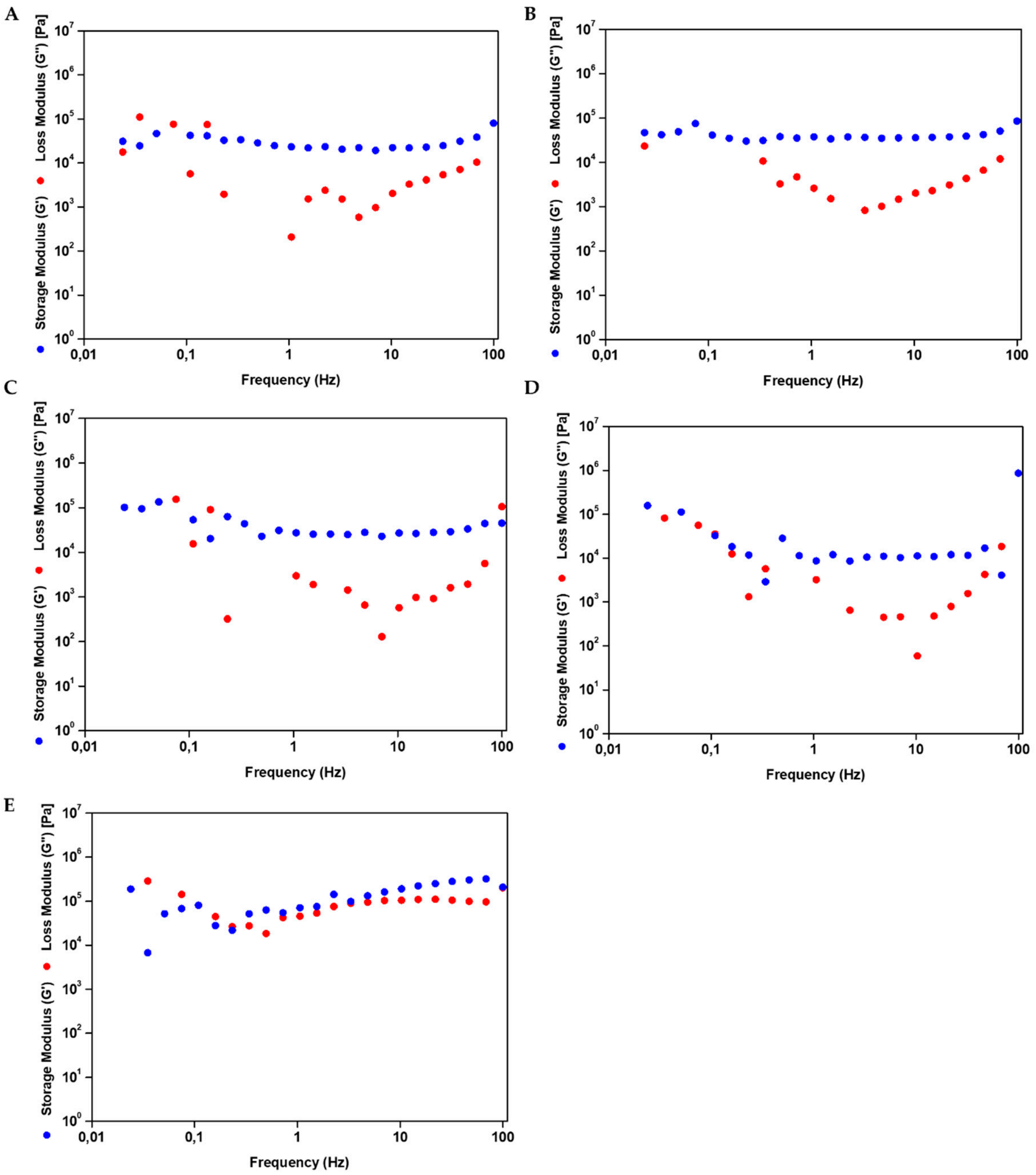

Figure S7. Frequency dependence of the shear elastic (G′) and loss (G′′) moduli for the hydrogels in study: (A) **1c** (0.4 wt%), (B) **1e** (0.5 wt%), (C) **2b** (0.4 wt%), (D) **2c** (0.4 wt%) and (E) **2d** (0.4 wt%).

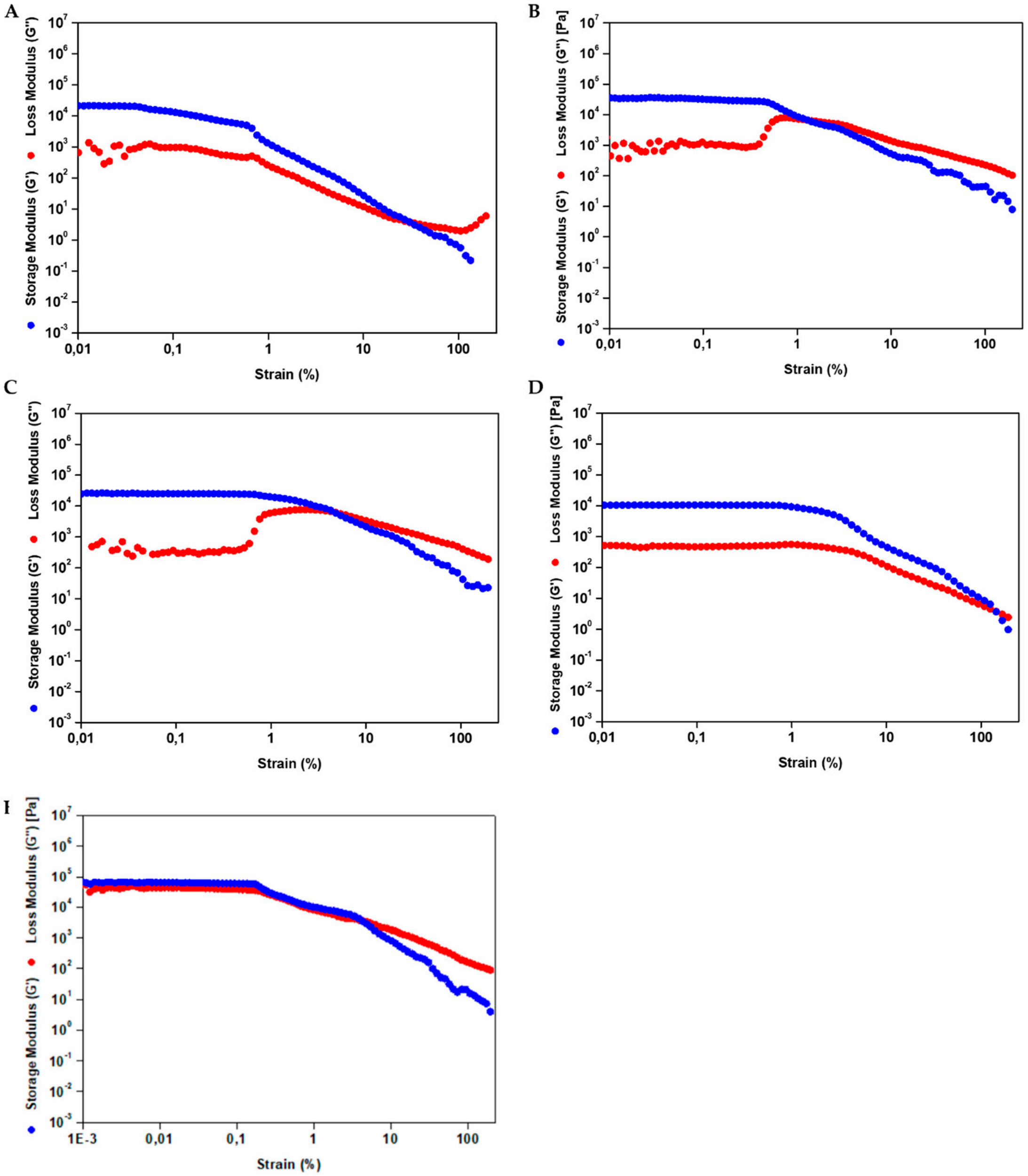

Figure S8. Strain dependence of the shear elastic (G’) and loss (G’’) modulus for hydrogels under study: (A) **1c** (0.4 wt%), (B) **1e** (0.5 wt%), (C) **2b** (0.4 wt%), (D) **2c** (0.4 wt%) and (E) **2e** (0.4 wt%).

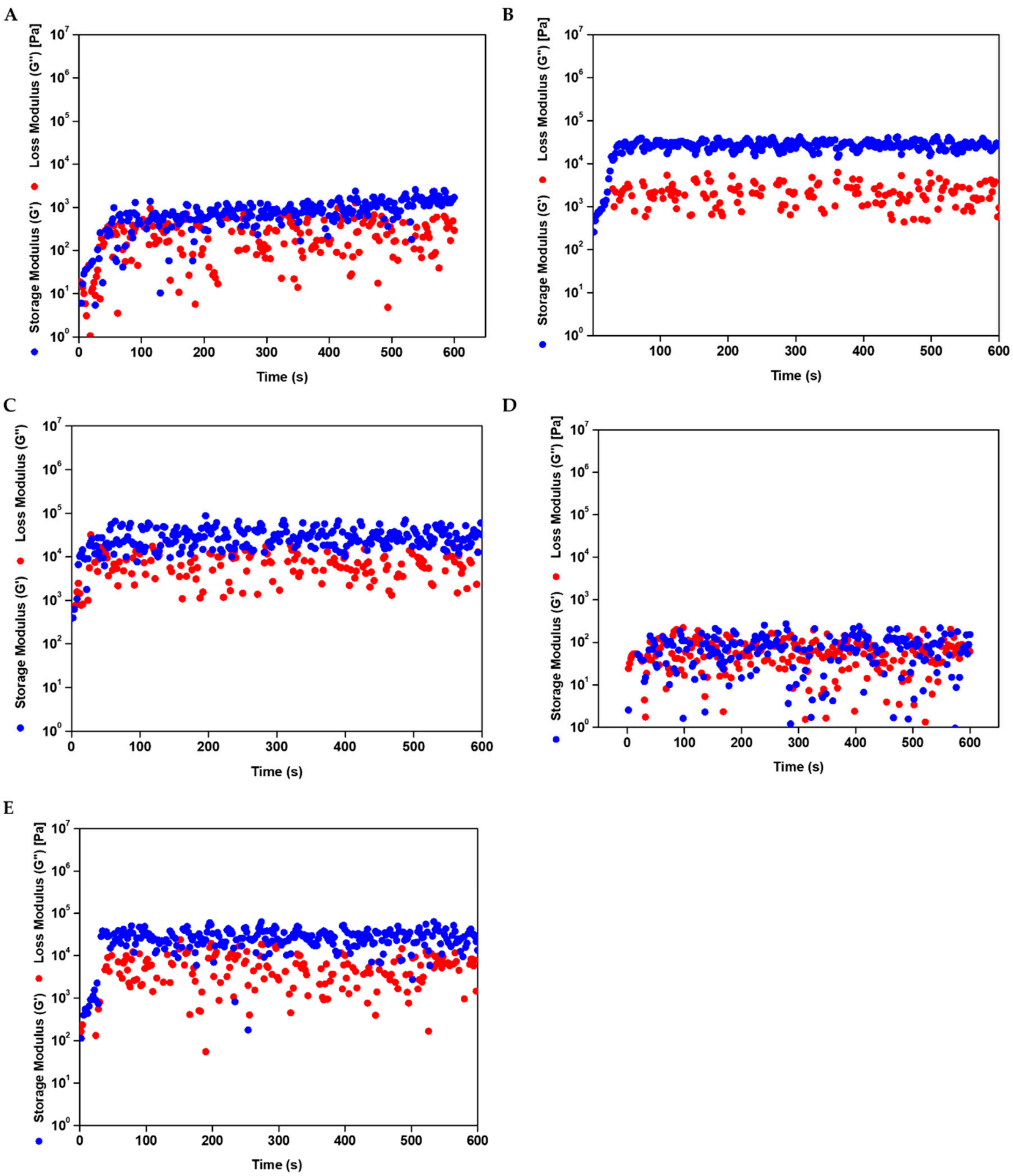

Figure S9. Elastic and viscous modulus during the second kinetic process of gelation for hydrogelators under study: (A) **1c**, (B) **1e**, (C) **2b**, (D) **2c** and (E) **2d**.

6. Drug Delivery Assays

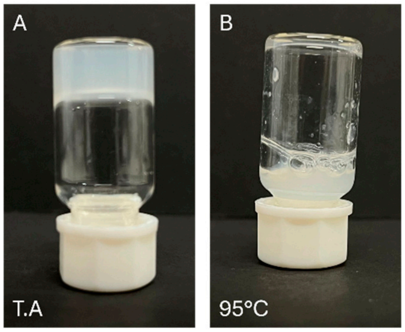

Figure S10. Sol-gel transition temperature of hydrogel **2a**. A) Hydrogel at room temperature. B) hydrogel after heating at 95 ºC.

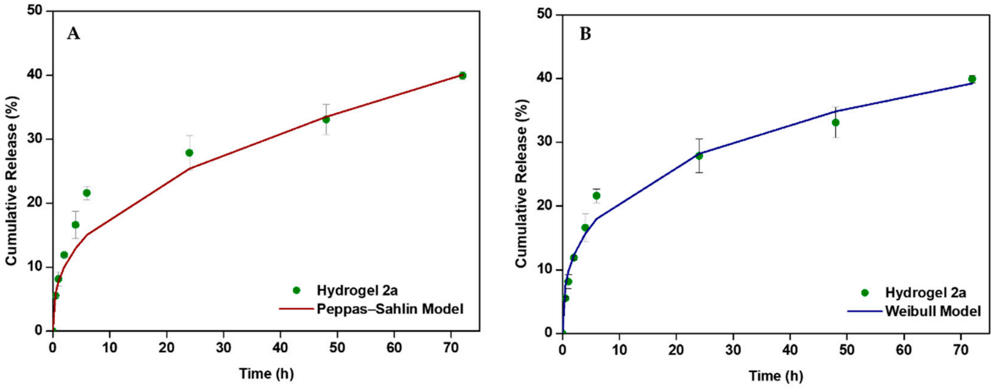

Figure S11. Cumulative release of MTX from hydrogel **2a** fitted to the Peppas-Sahlin Model (A) and Weilbull Model (B). Results presented as mean and standard deviation of three replicate assay.

Table S1. Parameters obtained by fitting the release profiles of drug-load hydrogel **2a** to the Peppas-Sahlin Model and Weilbull Model, with the respective coefficients of determination (R^2).

Peppas-Sahlin Model				Weibull Model		
K_1	K_2	m	R^2	*b*	*a*	R^2
0.0770	0.0000120	0.370	0.984	0.371	0.102	0.989

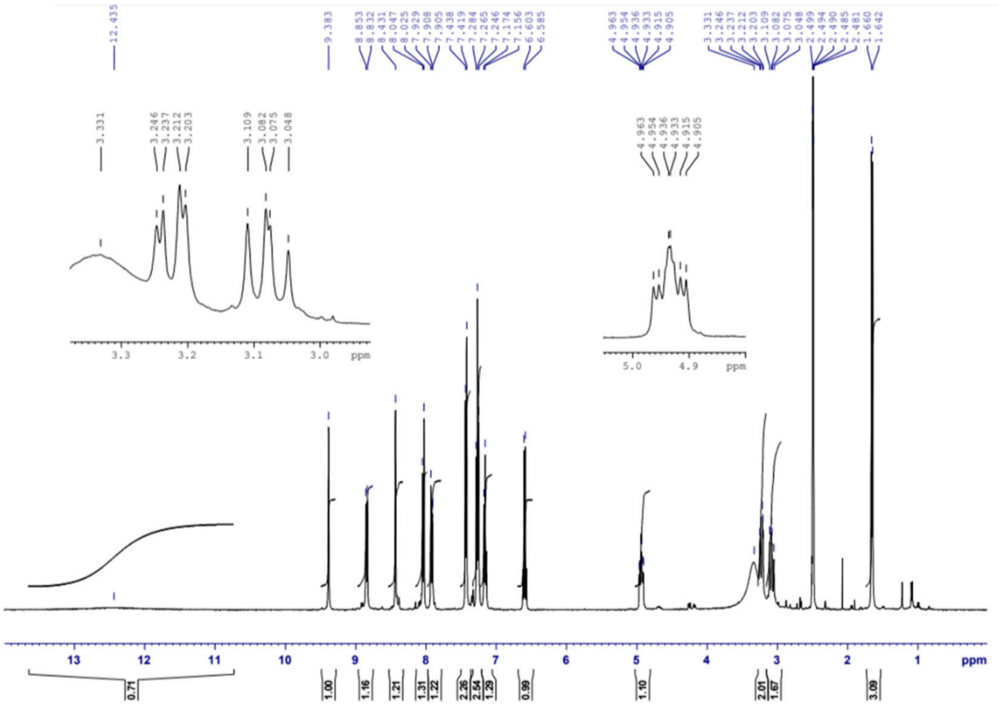

Figure S12. ^{1}H NMR spectrum of compound **1a** in DMSO-d_6.

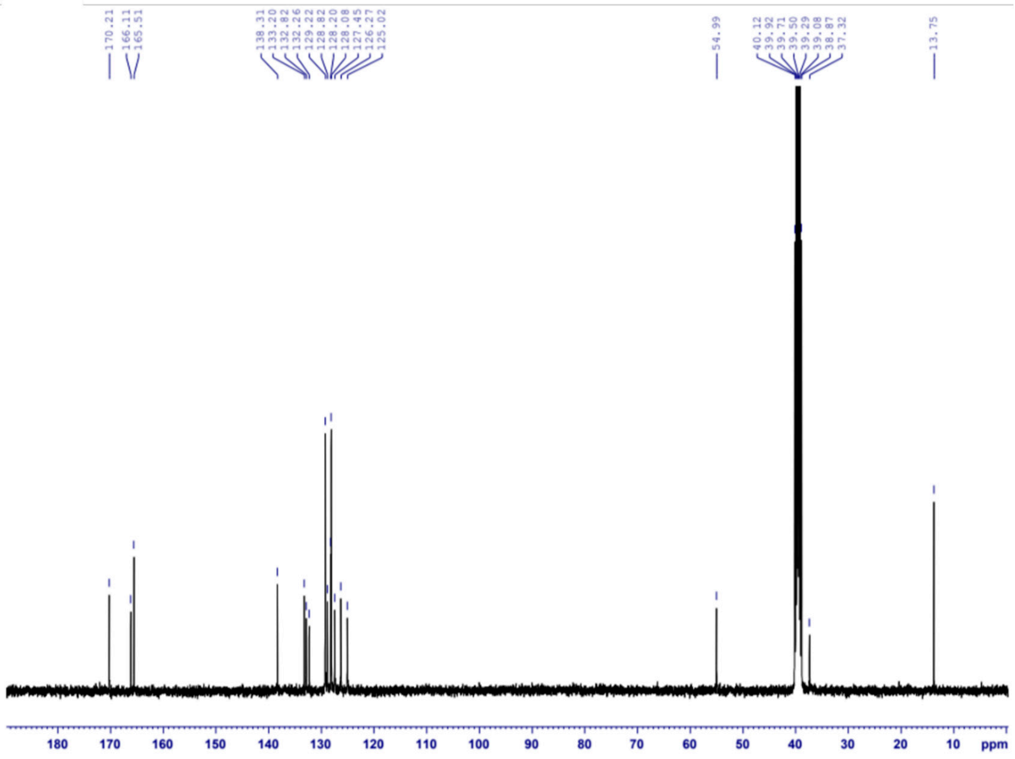

Figure S13. ^{13}C NMR spectrum of compound **1a** in DMSO-d_6.

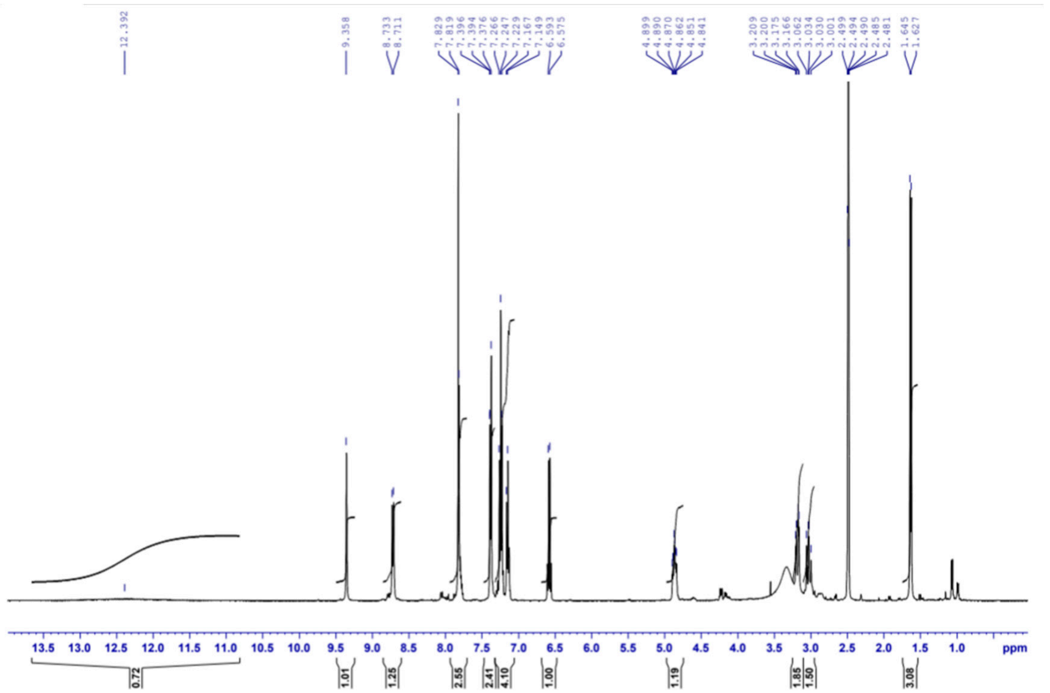

Figure S14. ^{1}H NMR spectrum of compound **1b** in DMSO-d_6.

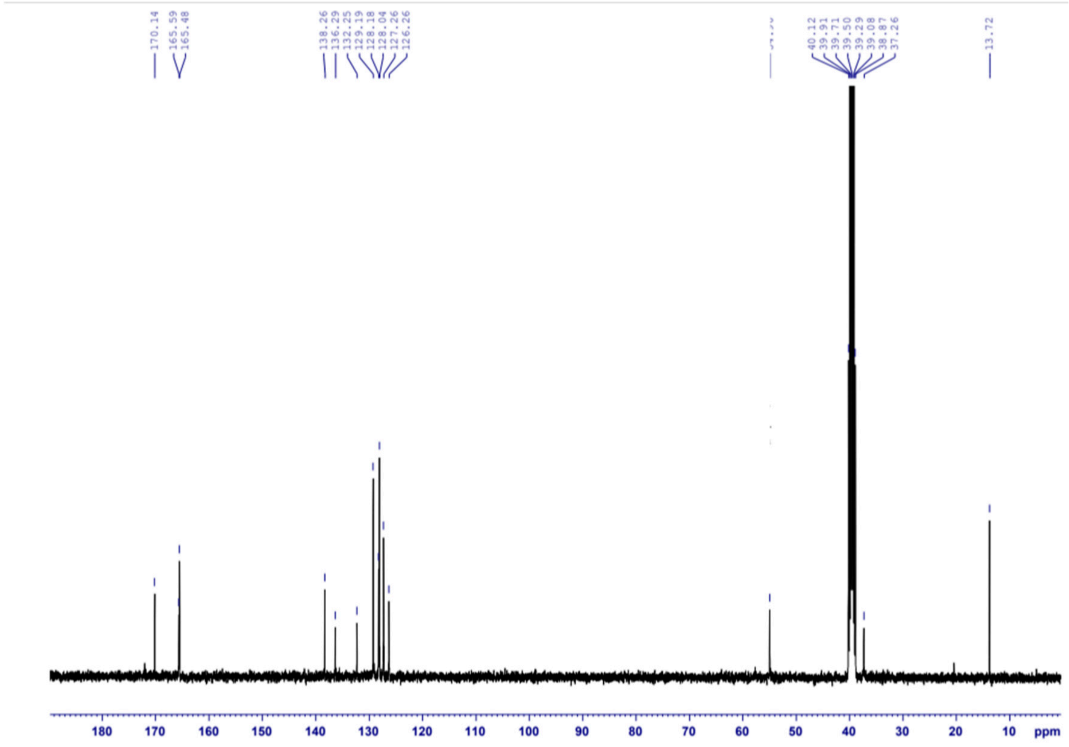

Figure S15. ^{13}C NMR spectrum of compound **1b** in DMSO-d_6.

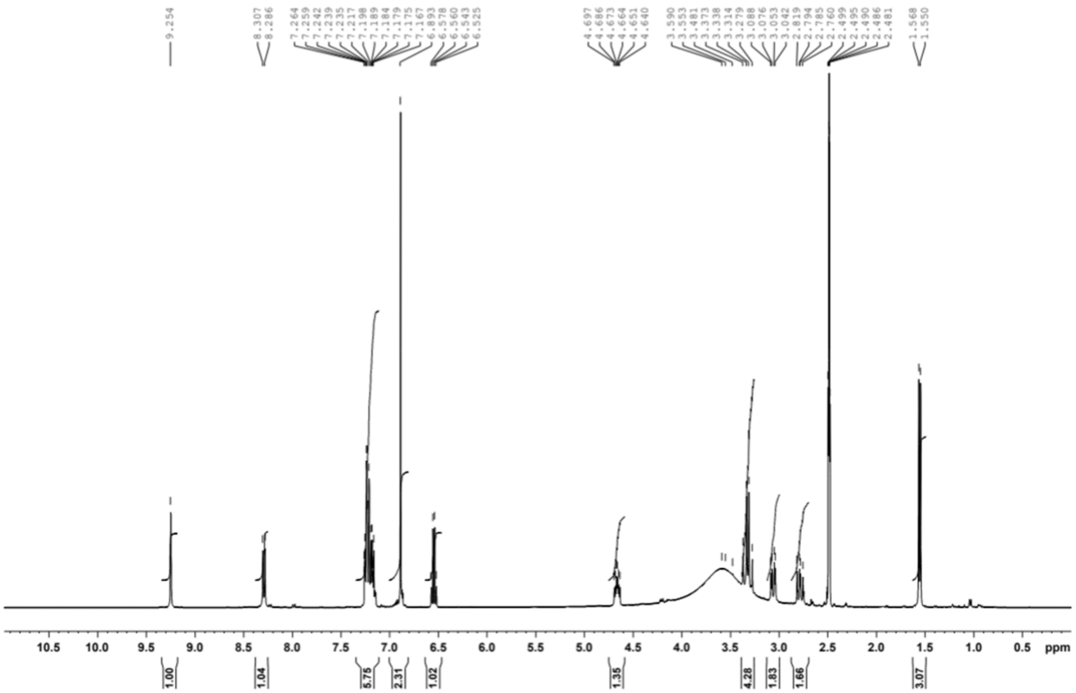

Figure S16. ^{1}H NMR spectrum of compound **1c** in DMSO-d6.

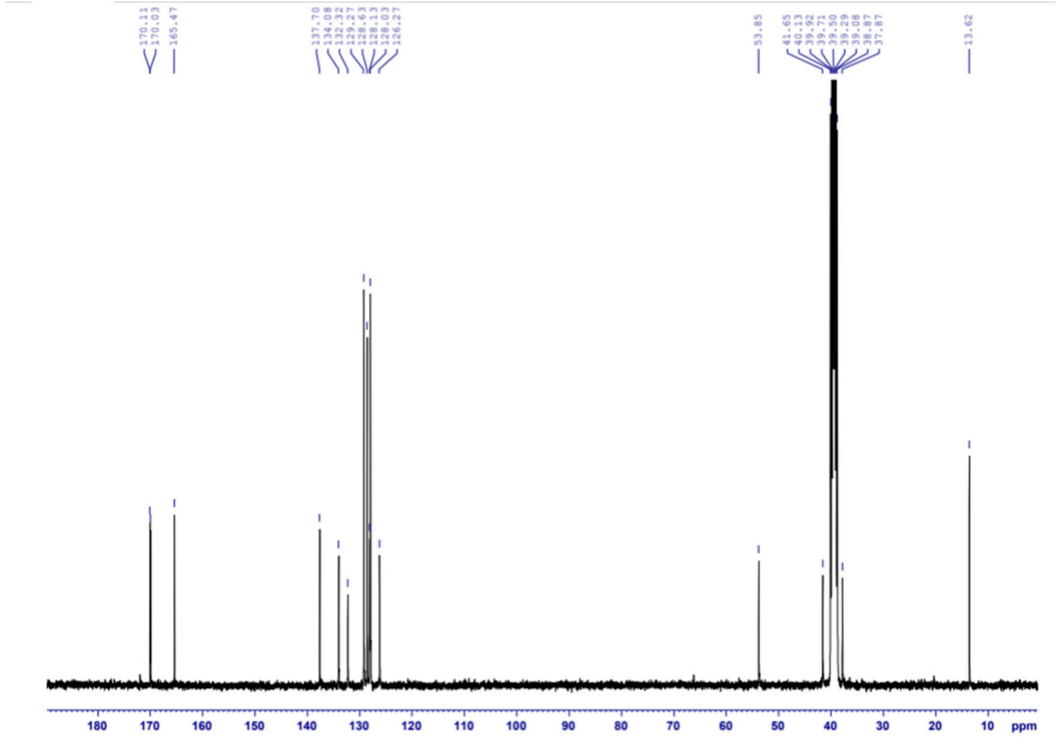

Figure S17. ^{13}C NMR spectrum of compound **1c** in DMSO-d6.

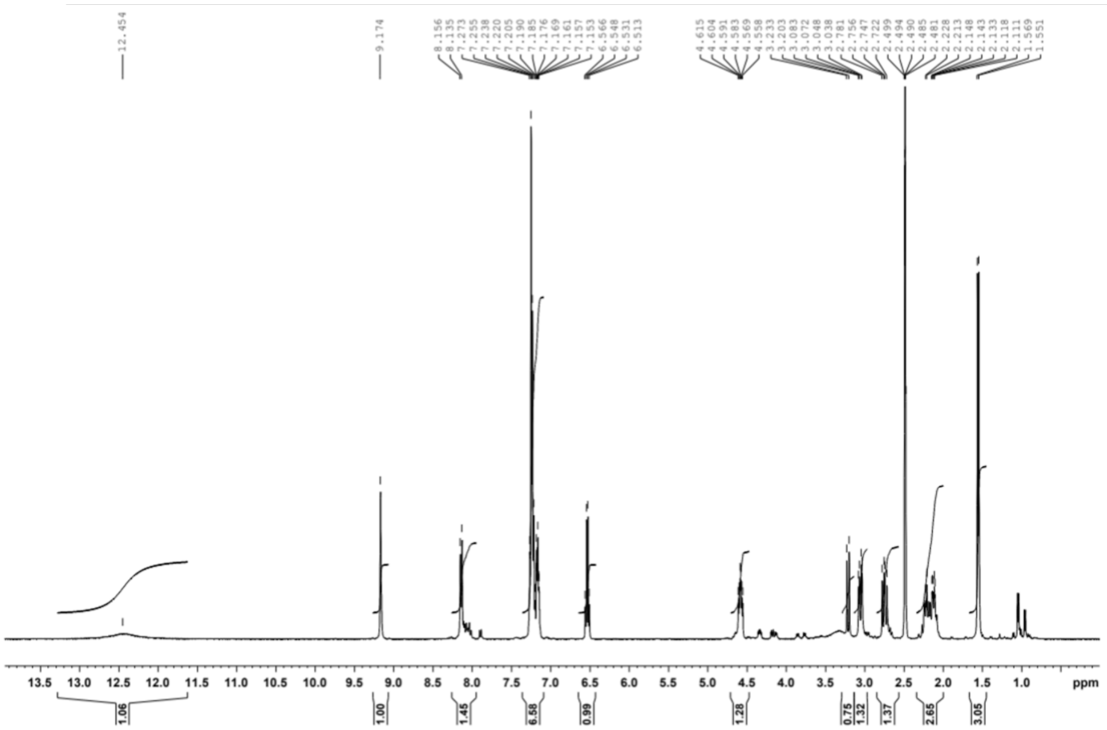

Figure S18. ^{1}H NMR spectrum of compound **1d** in DMSO-d_6.

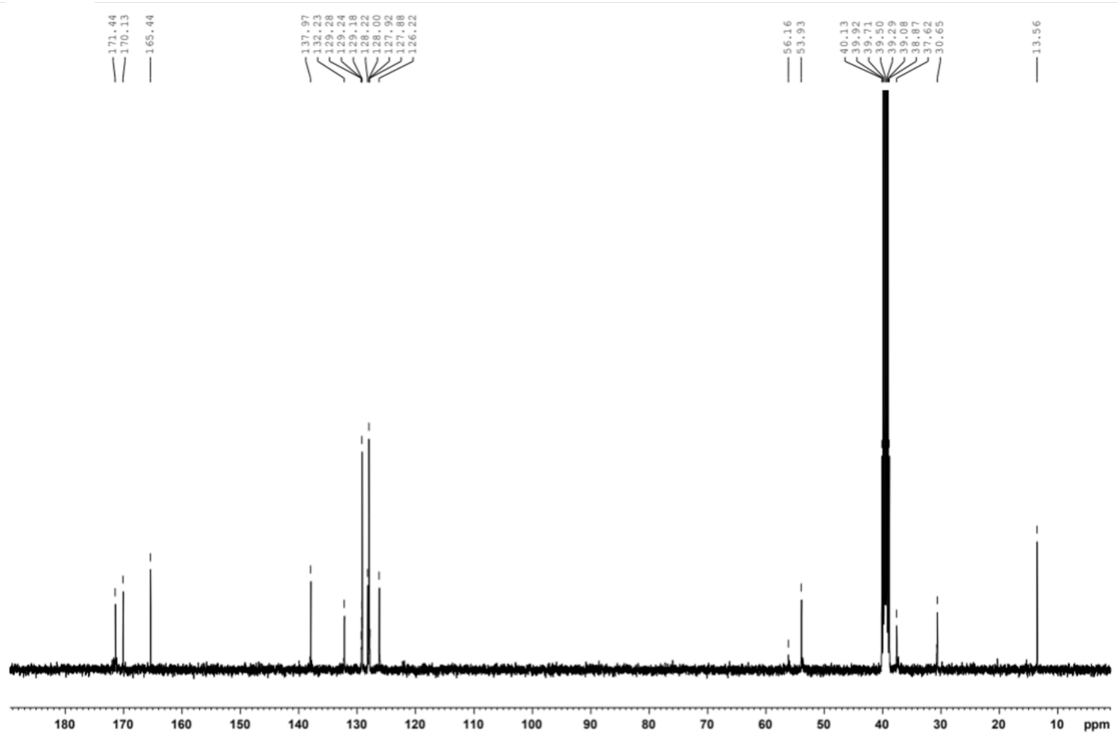

Figure S19. ^{13}C NMR spectrum of compound **1d** in DMSO-d_6.

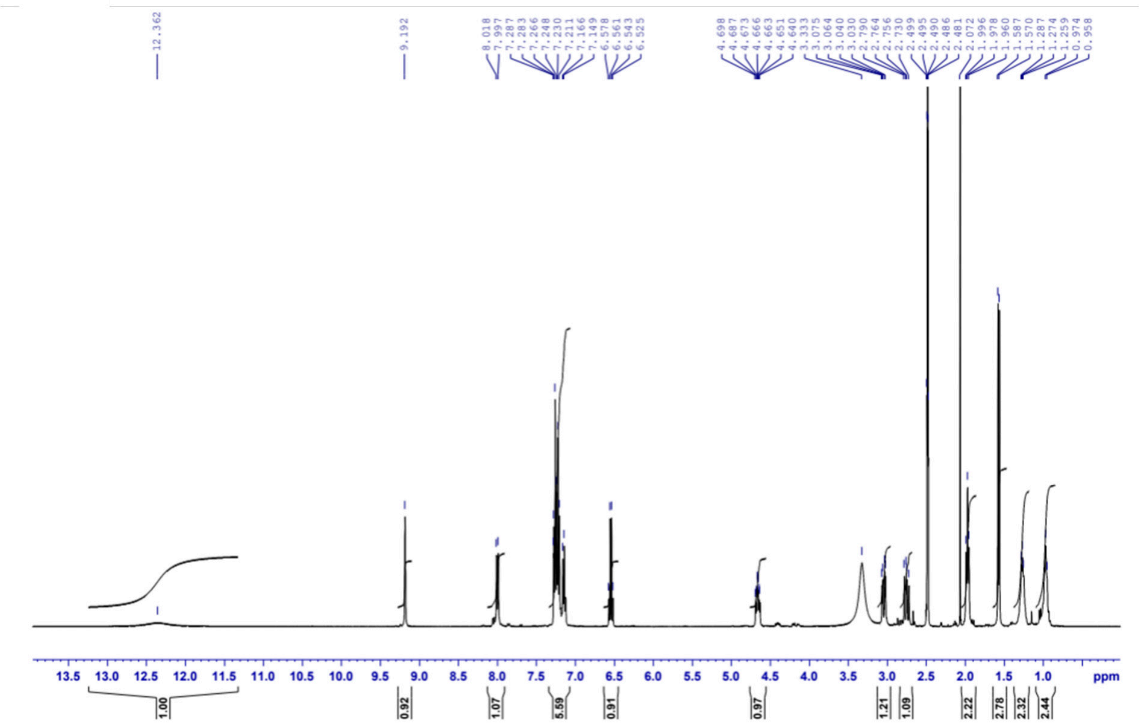

Figure S20. ^{1}H NMR spectrum of compound **1e** in DMSO-d_6.

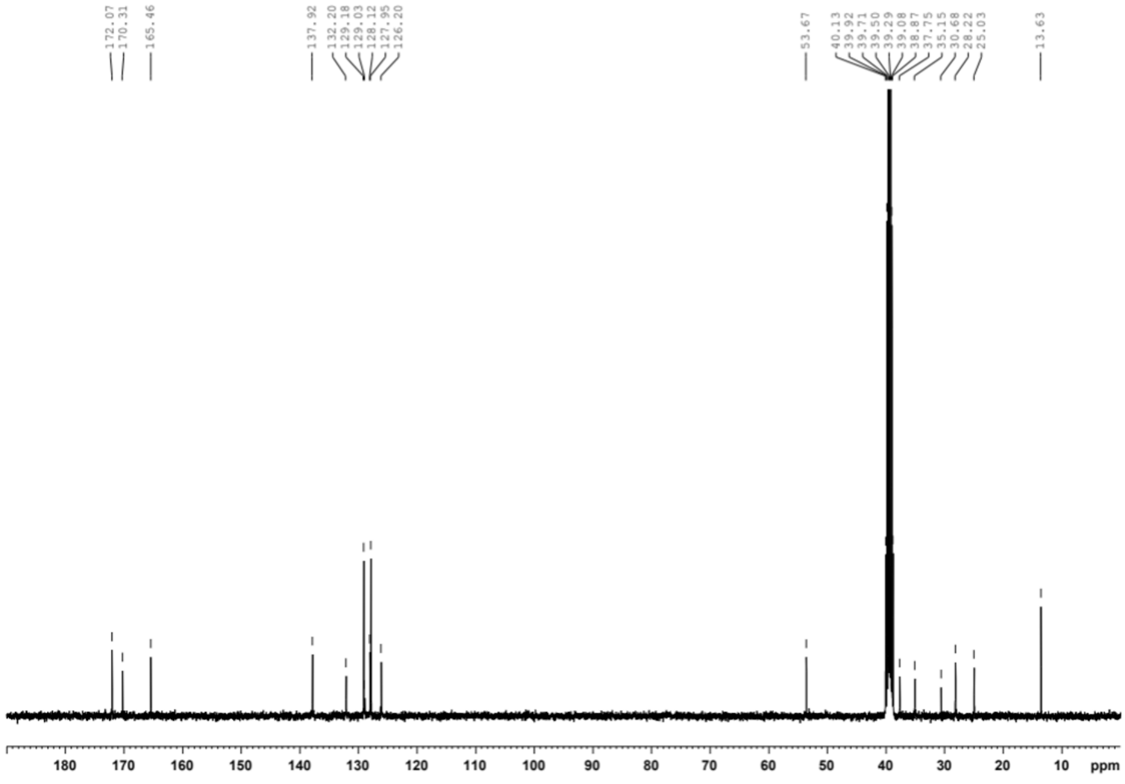

Figure S21. ^{13}C NMR spectrum of compound **1e** in DMSO-d_6.

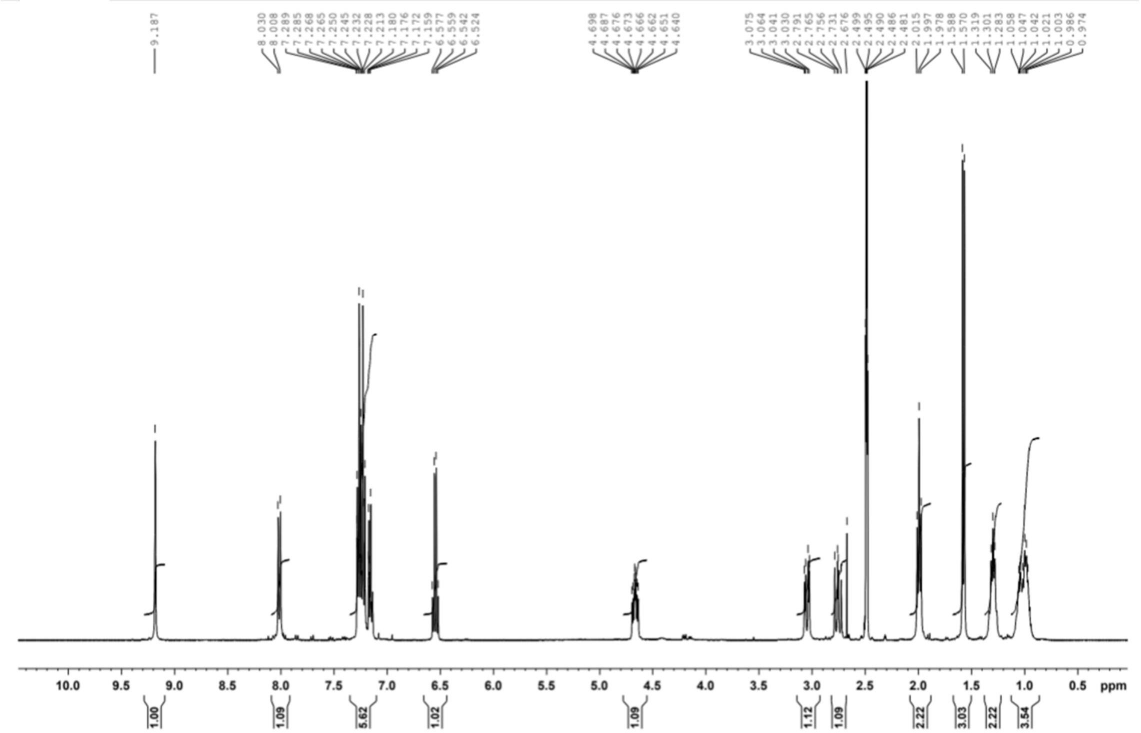

Figure S22. ^{1}H NMR spectrum of compound **1f** in DMSO-d6.

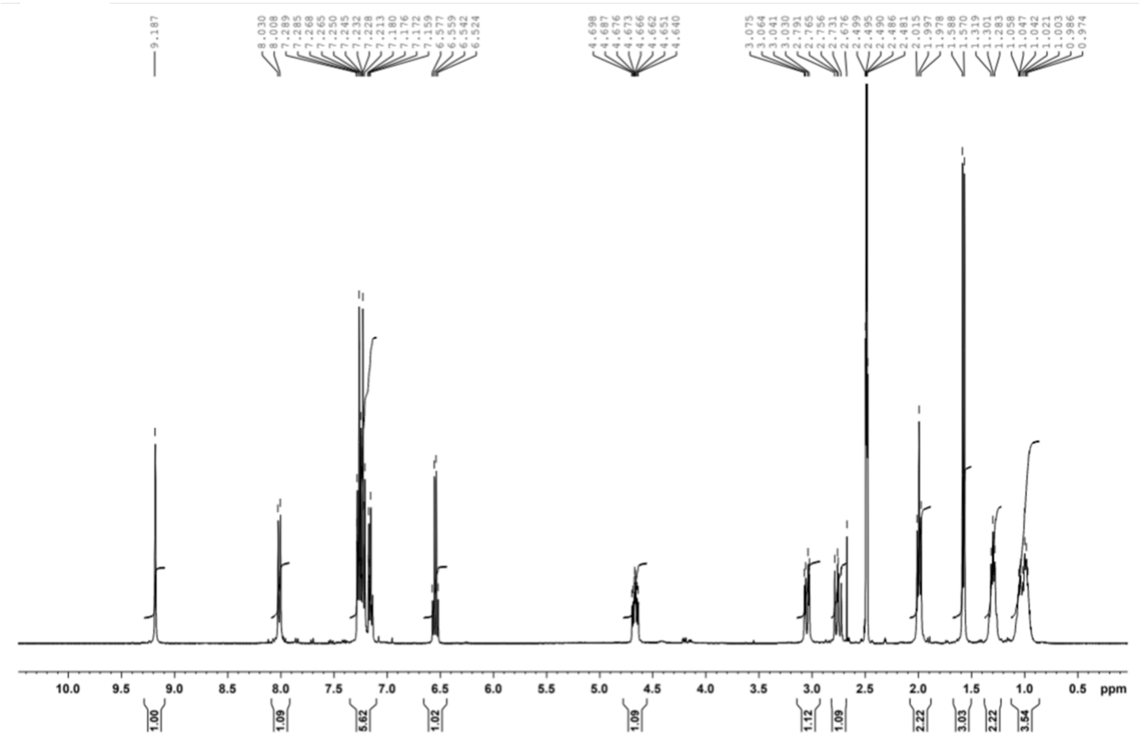

Figure S23. ^{13}C NMR spectrum of compound **1f** in DMSO-d6.

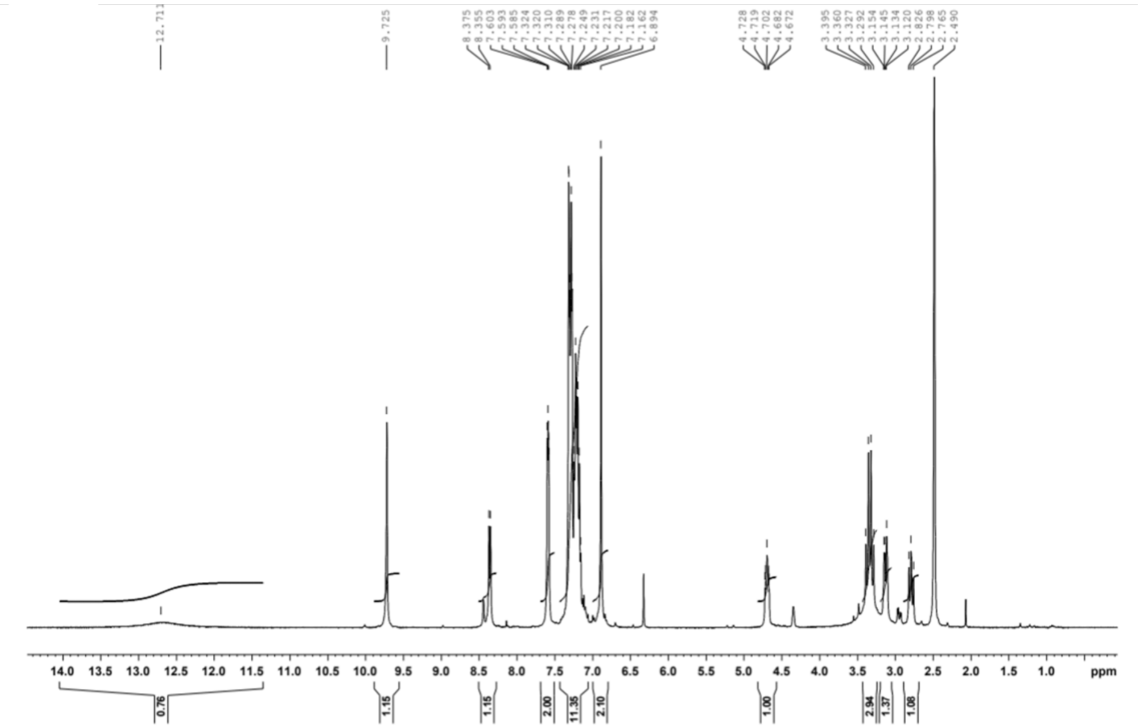

Figure S24. ^{1}H NMR spectrum of compound **2a** in DMSO-d_6.

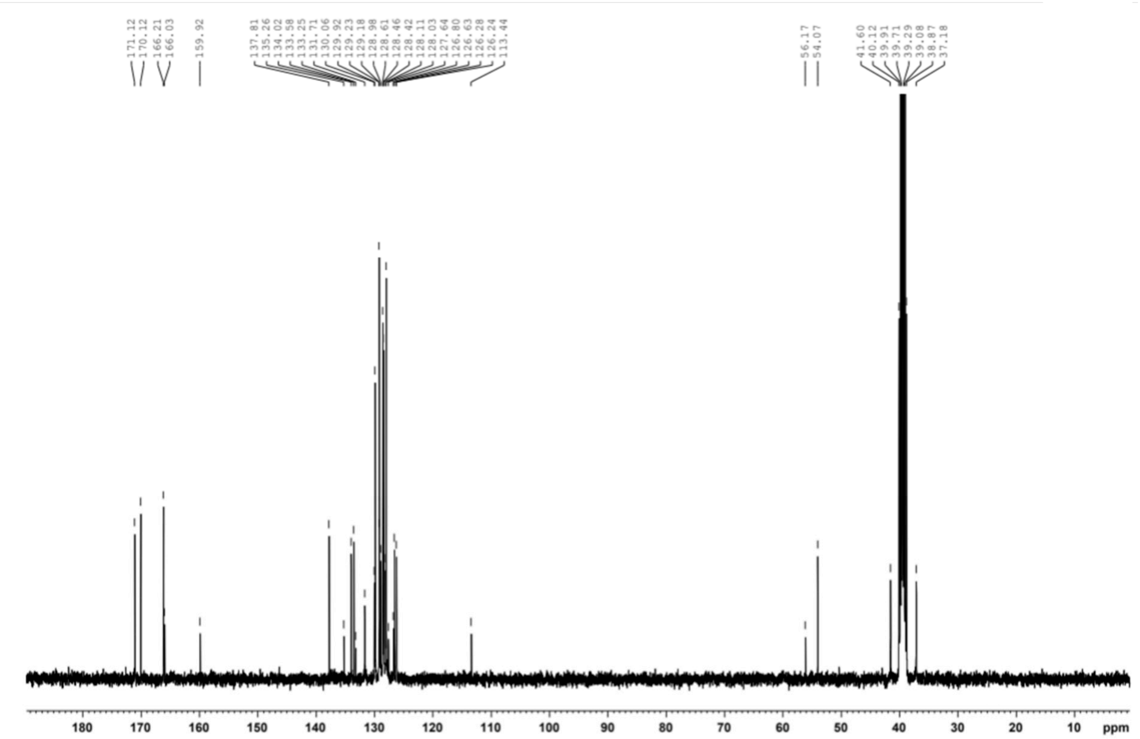

Figure S25. ^{13}C NMR spectrum of compound **2a** in DMSO-d_6.

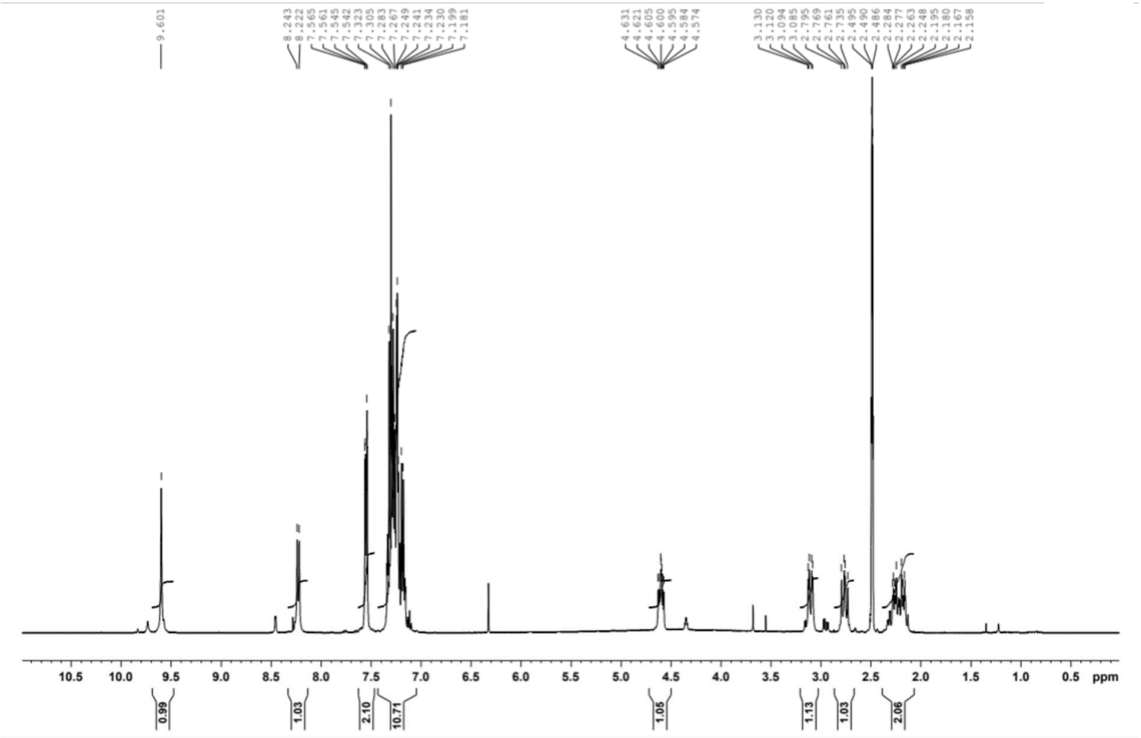

Figure S26. ^{1}H NMR spectrum of compound **2b** in DMSO-d$_6$.

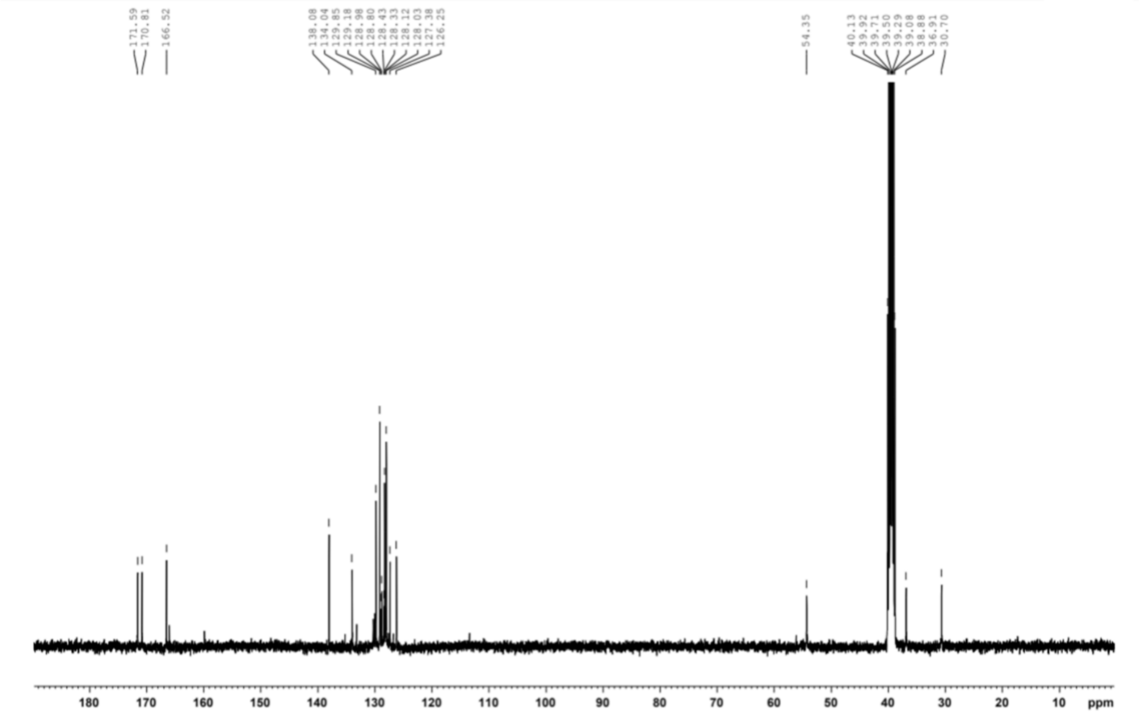

Figure S27. ^{13}C NMR spectrum of compound **2b** in DMSO-d$_6$.

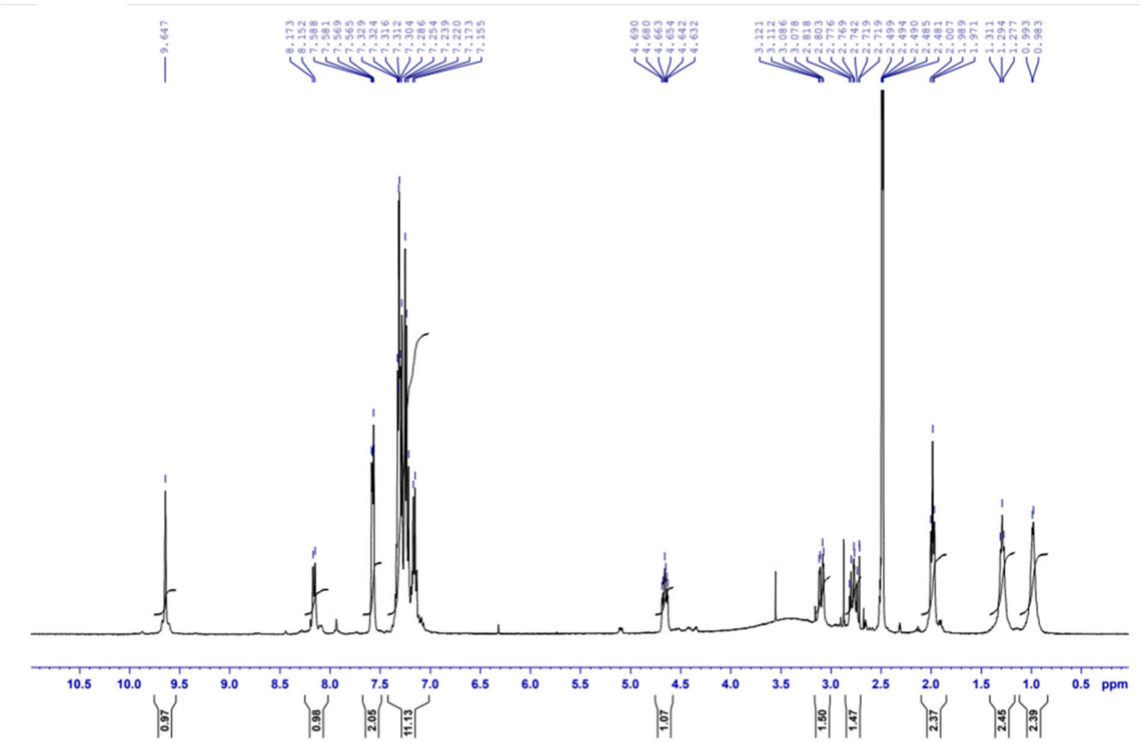

Figure S28. ^{1}H NMR spectrum of compound **2c** in DMSO-d_6.

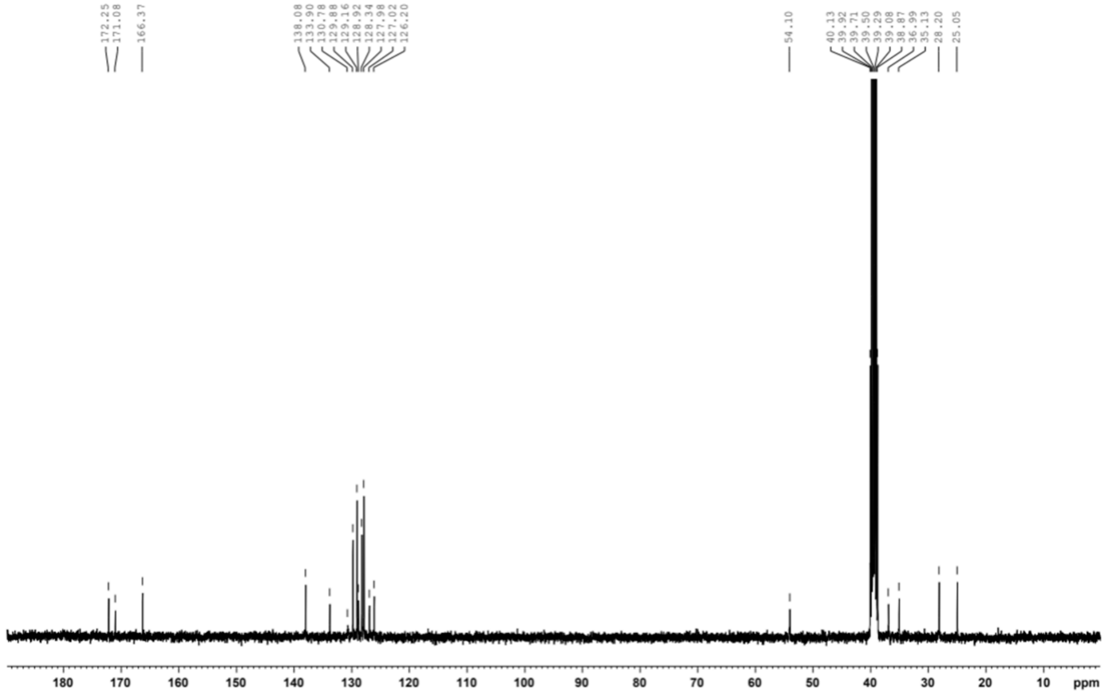

Figure S29. ^{13}C NMR spectrum of compound **2c** in DMSO-d_6.

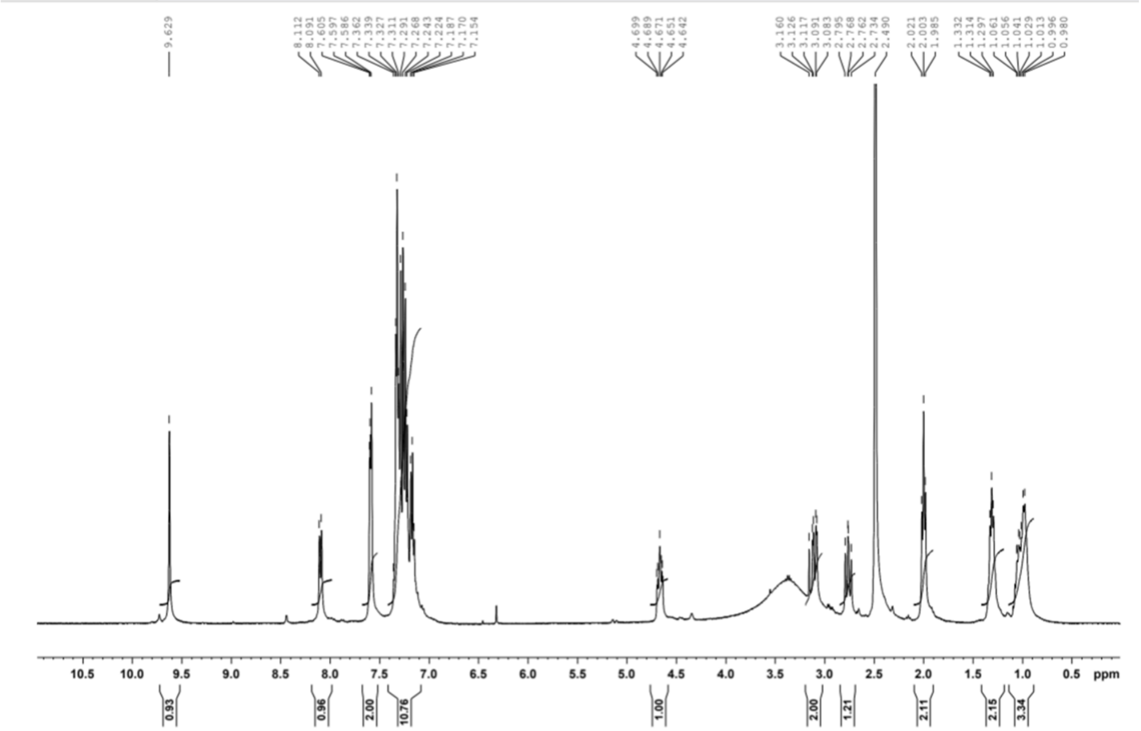

Figure S30. ^{1}H NMR spectrum of compound **2d** in DMSO-d_6.

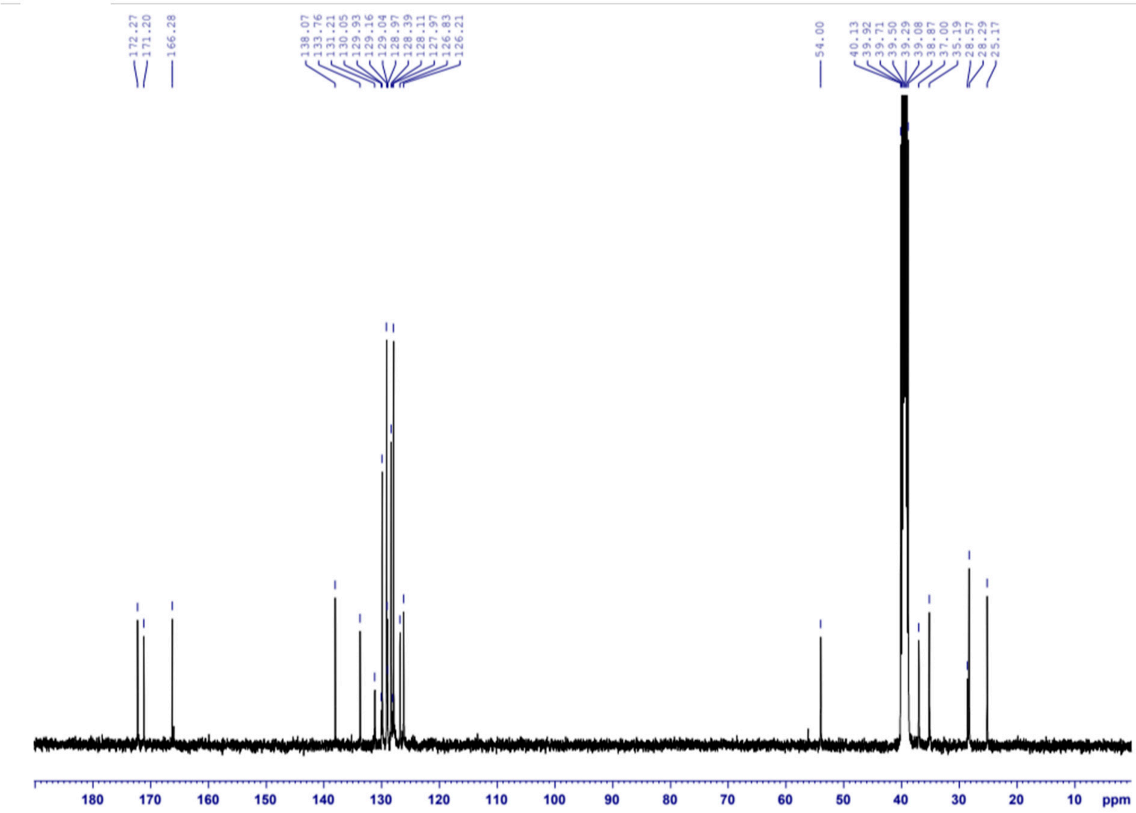

Figure S31. ^{13}C NMR spectrum of compound **2d** in DMSO-d_6.
